# Translating mechanobiology in osteoarthritis to biomaterial-informed therapeutic strategies

**DOI:** 10.1016/j.mbm.2026.100214

**Published:** 2026-09-23

**Authors:** Ziqi Gao, Yule Zheng, Hongxiang Mei, Juan Li

**Affiliations:** State Key Laboratory of Oral Diseases, National Center for Stomatology, National Clinical Research Center for Oral Diseases, West China Hospital of Stomatology, Sichuan University, Chengdu, 610041, China

**Keywords:** Osteoarthritis, Mechanobiology, Chondrocytes, Hydrogel, Nanomaterial

## Abstract

Osteoarthritis (OA) is the most prevalent degenerative joint disease worldwide, affecting approximately 250 million individuals. Mechanical imbalance serves as both its primary driving force and pathological hallmark. Mechanobiology-based interventions remain constrained by an insufficient understanding of how chondrocytes perceive and adapt to aberrant mechanical environments. Here, we review the cascading mechanisms linking mechanical imbalance to cellular dysfunction in OA and emerging therapeutic strategies targeting these mechanobiological processes. At the biomechanical level, joint mechanical imbalance encompasses both abnormal exogenous loading (trauma, obesity, joint deformity) and alterations in extracellular and pericellular matrix properties, with spatial heterogeneity and temporal dynamics throughout disease progression. At the cellular level, chondrocytes perceive mechanical signals through primary mechanosensors, including mechanosensitive ion channels (Piezo1/2, TRPV4), integrin–focal adhesion complexes, and primary cilia. These signals initiate Ca^2+^ signaling and cytoskeletal remodeling and converge on signaling and transcriptional regulators, including NF-κB, Wnt/β-catenin, YAP, and MAPK, ultimately contributing to cell death, cellular senescence, matrix degradation, and sustained inflammation. From a therapeutic perspective, we summarize mechanobiologically informed strategies, including macromechanical modulation, signaling regulation, and advanced biomaterial platforms for mechanical support, viscoelastic regulation, lubrication, drug delivery, and immunomodulation, aimed at restoring mechanical homeostasis and supporting tissue regeneration.

## Graphical abstract

**Figure:**
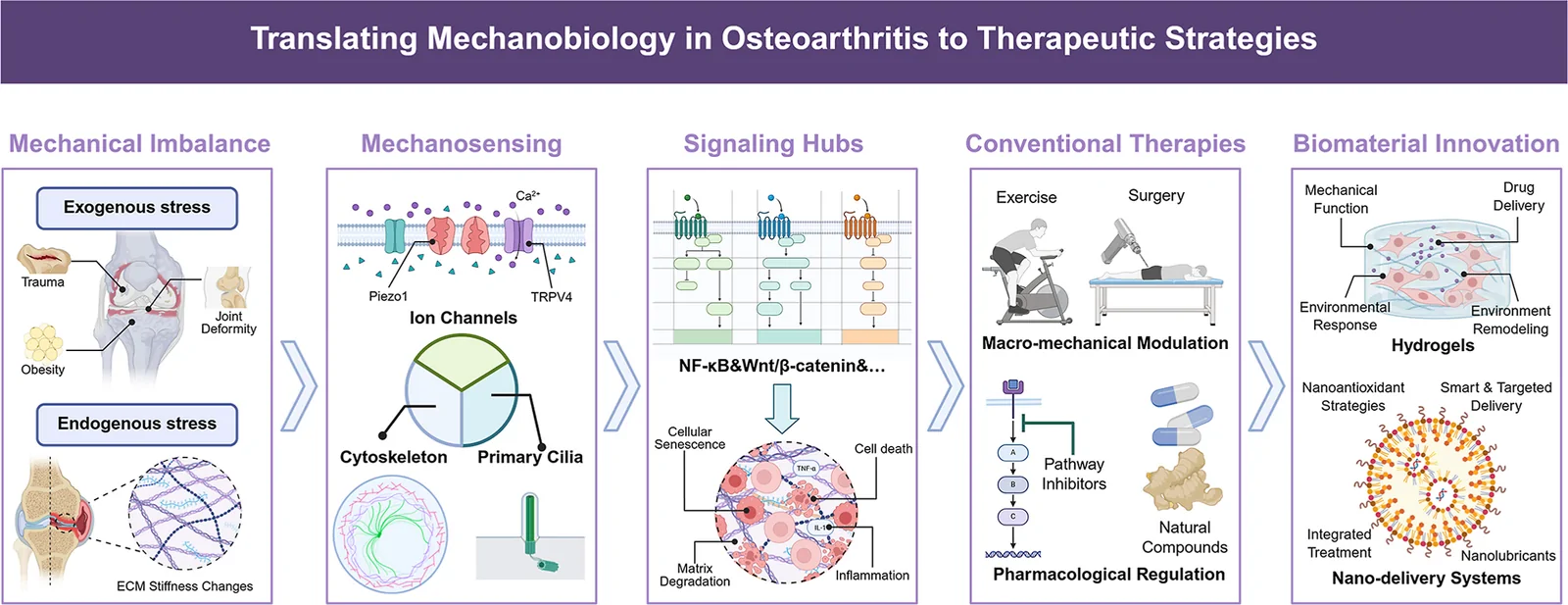

## 1 Introduction

OA is the most prevalent joint disease worldwide, affecting approximately 250 million individuals. This degenerative condition manifests as pain, joint stiffness, and mobility loss, profoundly affecting patients' quality of life.^1^ Current clinical practice guidelines recommend exercise therapy and weight management as first-line treatments for OA. These interventions have demonstrated efficacy in alleviating pain, optimizing joint loading, and improving patients' quality of life.^2^, ^3^, ^4^ Maintaining physiological mechanical loading is essential for joint health, whereas OA is accelerated by mechanical overload, including that associated with obesity, excessive exercise, and trauma.^5^^,^^6^ These clinical observations present challenges for biomechanics-based OA treatment: how can biomechanical responses be effectively harnessed to establish a therapeutic microenvironment that facilitates joint repair and reconstruction?

The joint mechanical microenvironment is shaped by exogenous mechanical loading and the intrinsic mechanical properties of the extracellular matrix (ECM) and pericellular matrix (PCM), which are critical regulators of joint homeostasis. Exogenous mechanical loading primarily arises from body weight, movement, joint kinematics, and external forces. Physiological loading promotes ECM synthesis and turnover, thereby maintaining joint function.^7^^,^^8^ Conversely, excessive or aberrant loading, such as that caused by overuse or trauma, triggers inflammatory responses and cartilage degeneration.^9^^,^^10^ Intrinsic matrix-derived mechanical cues arise from ECM components, including proteoglycans (PGs), collagen fibers, and other matrix proteins.^11^^,^^12^ Alterations in ECM biophysical properties, such as stiffness, viscoelasticity, permeability, and fibrillar organization, modify the transmission of joint loading and the cell-scale mechanical states experienced by chondrocytes. These changes may impair chondrocyte mechanosensation and mechanotransduction, ultimately causing cellular dysfunction and cartilage degeneration.^13^, ^14^, ^15^ During OA progression, both exogenous loading and matrix mechanical properties undergo dynamic alterations, complicating the assessment of mechanical environmental effects and challenging the development of biomechanics-based therapeutic interventions.

This review comprehensively examines alterations in mechanical loading and matrix mechanical properties during OA pathogenesis, focusing on cartilage responses to aberrant mechanical environments. We further summarize current biomechanics-based therapeutic strategies to provide insights into future directions for OA management. By integrating pathological mechanical inputs, cell-scale mechanical states, mechanotransduction, and biomaterial-informed interventions, this review aims to bridge fundamental biomechanical research with clinical translation and support the development of innovative therapeutic paradigms for OA.

## 2 Mechanical imbalance in OA: from macro to micro

### 2.1 Multiscale definition of physiological loading and mechanical overload

The mechanical environment of knee cartilage is spatially heterogeneous and depends on activity, alignment, body mass, tissue properties, hydration, loading rate, and loading duration. Therefore, physiological loading and mechanical overload cannot be defined by a single universal stress threshold. In this review, physiological loading refers to local mechanical stimulation that can be accommodated by cartilage and chondrocyte homeostatic mechanisms, whereas mechanical overload refers to focal or excessive stress/strain associated with chondrocyte injury or matrix degeneration. At the tissue scale, a maximum shear strain of approximately 15–30% may represent a transitional risk range, whereas values of approximately 30% or higher have been associated with cartilage matrix degeneration in ex vivo and finite-element models.^16^ At the cellular scale, AFM–FEM studies suggest that whole-cell deformation of approximately 80–85% and an apparent principal membrane strain exceeding 1.31 may indicate exhaustion of membrane-buffering capacity and increased risk of pathological mechanotransduction.^17^ These values are model-based reference ranges rather than universal clinical thresholds.

### 2.2 Abnormal exogenous mechanical loading in OA

During daily activities and locomotion, articular cartilage experiences exogenous mechanical loading, including compression, tension, shear, and torsion.^18^^,^^19^ These loads mainly arise from body weight, gait and movement patterns, joint kinematics, and posture. They are dynamic, multidirectional, and repetitive.^18^ Physiological loading promotes ECM anabolism, supports chondrocyte homeostasis, and is essential for preserving cartilage structural integrity and function.^20^^,^^21^

OA development is frequently associated with abnormal exogenous mechanical loading, including overload and uneven load distribution.^10^^,^^22^, ^23^, ^24^, ^25^ Using clinical imaging—computed tomography (CT) and magnetic resonance imaging (MRI)—combined with finite element analysis, studies have shown that weight gain, joint deformities (e.g., varus deformity), ligamentous or tendinous injuries (e.g., Calcaneofibular ligament injury), and malunited fractures can induce abnormal joint load distribution^26^, ^27^, ^28^, ^29^, ^30^, ^31^, ^32^ (Table 1). To be more specific, (1) chronic obesity elevates joint contact loads throughout the gait cycle. This sustained mechanical overloading accelerates cartilage degradation, particularly during the stance phase when contact loading peaks in the medial knee compartment.^33^ (2) Traumatic injuries alter load distribution, particularly through damage to bone and ligament structures. (3) Tibial fracture–related deformities produce focal loading concentrations in specific regions. (4) In the knee, varus deformity concentrates load in the medial tibiofemoral compartment, whereas valgus concentrates load laterally. Both patterns increase cartilage damage in peak-loading regions.^34^, ^35^, ^36^ (5) Anterior cruciate ligament (ACL) injury shifts loading primarily to the posteromedial tibial plateau, with reduced loading in the anteromedial region. With increasing duration of ACL deficiency, posteromedial loading concentration further intensifies.^37^

**Table 1:** Summary of characteristics of abnormal exogenous loading environments in knee OA.

Source of loading (injury/intervention)	Site of loading concentration	Site of injury/lesion
**Chronic obesity**	Medial compartment of the knee	Increased cartilage wear in the medial compartment
**Medial knee deformity**	Medial knee plateau	Medial plateau cartilage damage
**Knee valgus deformity**	Lateral plateau of the knee	Lateral Plateau Cartilage Injury
**ACL Injury**	Posteromedial tibial plateau region	Osteoarthritic lesions in the posterior medial region
**Proximal fibular osteotomy (PFO)**	Loading transfer from anteromedial to posteromedial with increased loading on the lateral compartment	NA
**Open wedge high tibial osteotomy (OWHTO)**	Medialmost region of tibial plateau	NA
**Closed wedge high tibial osteotomy (CWHTO)**	Second lateral region of the tibial plateau	NA

Surgical interventions can intentionally modulate joint load distribution. On the one hand, proximal fibular osteotomy (PFO) shifts knee joint loading from the anteromedial to the posteromedial tibial plateau, significantly reducing peak loading in the medial compartment while increasing peak loading laterally.^38^ Yet, Open-wedge high tibial osteotomy (OWHTO) concentrates loading in the most medial region of the tibial plateau, whereas closed-wedge HTO (CWHTO) shifts loading to a more lateral region of the plateau, suggesting that therapeutic benefit can be achieved by surgically altering load distribution.^39^ For example, elevated medial compartment loading corresponds to clinical findings of cartilage degeneration and osteophyte formation within the medial knee compartment^40^^,^^41^ (Table 1).

In animal models of OA, the spatial correspondence between lesion location/severity and mechanical loading remains evident. However, different models exhibit distinct patterns of load distribution. For example, in monosodium iodoacetate (MIA) models, cartilage damage is relatively uniform, and contact-loading distribution is homogeneous, with overall loading levels being lower.^42^ In contrast, both ACLT and DMM increase medial compartment loading, but with differing loading patterns and lesion distributions. ACLT shifts contact loading toward the anteromedial tibial plateau, resulting in pronounced damage in anterior regions.^43^ Unlike ACLT, DMM, induced by transection of the medial meniscotibial ligament, destabilizes the medial meniscus and concentrates loading within the central weight-bearing zone of the medial tibial plateau and medial femoral condyles.^44^

In summary, abnormal distribution of exogenous mechanical loading plays a key role in the progression of OA. Both clinical and preclinical studies show that joint regions with contact-loading concentration spatially correlate with the severity of osteoarthritic lesions. This underscores the central role of mechanical factors in OA pathophysiology and provides a rationale for clinical intervention. Modulating and redistributing exogenous joint loading through surgical procedures may enable more precise, individualized treatment strategies for patients with OA.

### 2.3 Altered matrix mechanical properties and cell-scale mechanical states in OA

The ECM provides the material and structural basis of the chondrocyte mechanical microenvironment, and its principal components, including PGs and collagen fibers, are major determinants of cartilage mechanical properties.^11^^,^^45^ Cartilage ECM can be divided into PCM, territorial matrix, and interterritorial matrix. The PCM directly surrounds chondrocytes and serves as a critical interface between chondrocytes and the bulk cartilage matrix, playing a pivotal role in mechanosensation and mechanotransduction.^46^ Through their composition, architecture, and material properties, the ECM and PCM regulate the transmission and redistribution of joint loading and shape the tissue- and cell-scale mechanical states experienced by chondrocytes. These properties, including stiffness, viscoelasticity, stress relaxation, permeability, and fibrillar organization, influence local stress and strain distributions, fluid flow, and cellular deformation. Consequently, ECM biophysical properties regulate chondrocyte proliferation, differentiation, and metabolism, shape cellular mechanosensation and responses, and ultimately influence chondrocyte function and fate.^11^^,^^13^^,^^46^^,^^47^

Cartilage matrix stiffness undergoes complex multi-level alterations during OA progression, thereby modifying matrix load transfer and the mechanical states experienced by chondrocytes. At the macroscopic (millimeter) scale, overall cartilage stiffness typically declines, primarily owing to matrix component degradation and loss of mechanical integrity.^48^ However, focal surface stiffening may occur in overloaded regions and is associated with increased lysyl oxidase (LOX) expression and accumulation of advanced glycation end products (AGEs). These changes enhance enzymatic and non-enzymatic collagen cross-linking and stiffen specific ECM regions.^49^ At the microscopic (micrometer) scale, stiffness shows marked regional heterogeneity: superficial layers typically soften, whereas deep layers change minimally. The magnitude of stiffness reduction differs between the ECM and PCM. The apparent modulus of the ECM decreases by up to 45% (from 491 ± 112 kPa to 270 ± 76 kPa), whereas PCM stiffness declines by approximately 30-39% (from 137 ± 22 kPa to 96 ± 16 kPa). These differences may reflect the mechanical buffering by type VI collagen and fibronectin, which are abundant in the PCM.^50^ At the nanoscale (nanometer level), matrix stiffness may increase due to collagen fibril rearrangement and increased cross-linking.^51^ This spatiotemporal heterogeneity in multiscale matrix stiffness contributes to an imbalanced chondrocyte mechanical microenvironment during OA progression, disrupting matrix-cell mechanosignaling and impairing the balance between cartilage degeneration and repair.

Multi-scale differences in cartilage matrix stiffness can be attributed to multiple factors. To begin with, OA-associated alterations in the content and distribution of collagen and PGs reduce overall matrix stiffness. In contrast, collagen fibril reorganization and increased cross-linking can increase nanoscale stiffness.^51^ Furthermore, collagen architecture critically influences matrix stiffness. Under physiological conditions, collagen fibers are aligned parallel to the articular surface in the superficial zone and perpendicular to the surface in deeper zones, producing a depth-dependent stiffness gradient. In OA, this architectural disruption can cause locally increased fiber density and cross-linking, resulting in stiffening in some regions and softening in others.^52^ Finally, reported stiffness values are also affected by measurement modality and experimental conditions. For example, in indentation-based measurements, indenter geometry and indentation depth alter the contact area and deformation field, while imperfect probe-surface congruence on curved cartilage can introduce measurement uncertainty. AFM measurements are particularly sensitive to vibration and temperature fluctuations and sample only a limited area, whereas differences in acquisition time, sample collection, storage, and processing may further affect the stability and comparability of stiffness estimates.^53^ In summary, compositional alterations and collagen architectural remodeling drive multiscale changes in cartilage matrix stiffness during OA progression. Together with changes in other matrix properties, these alterations modify the transmission of joint loading and the local mechanical states perceived by chondrocytes, thereby contributing to aberrant mechanosensation and chondrocyte dysfunction.

### 2.4 Interaction between exogenous mechanical loading and matrix-derived mechanical cues

In OA, abnormal exogenous mechanical loading alters cartilage matrix mechanical properties.^45^^,^^46^ On the one hand, mechanical overload can induce global matrix softening: excessive loading upregulates matrix metalloproteinases (MMPs) and aggrecanases, promoting collagen and proteoglycan degradation and weakening ECM mechanical integrity.^20^ On the other hand, abnormal exogenous loading can also cause localized matrix stiffening. Excessive loading increases collagen cross-linking through enzymatic pathways (LOX-mediated) and non-enzymatic glycation by AGEs, enhancing fibril rigidity and thereby stiffening the ECM and PCM.^54^ Concurrently, nanoscale reorganization and reorientation of collagen fibrils may increase local matrix density and stiffness. Together, these processes drive focal matrix stiffening. The interaction between exogenous mechanical loading and matrix mechanical properties is pivotal in OA onset and progression, providing a mechanistic basis for mechanically informed therapeutic strategies.

The coupling between exogenous mechanical loading and matrix-derived mechanical cues profoundly affects chondrocyte biology. Abnormal loading directly alters the cell-scale mechanical states experienced by chondrocytes and secondarily modifies matrix properties, including stiffness, thereby establishing a closed-loop “loading-matrix-cell” feedback mechanism. Functionally, overload reduces chondrocyte proliferation, increases apoptosis, suppresses matrix anabolism, including type II collagen and proteoglycan synthesis, and markedly upregulates the expression of catabolic enzymes, including MMPs and ADAMTSs. Moreover, this interaction drives phenotypic shifts, including hypertrophic differentiation, activation of the senescence-associated secretory phenotype (SASP), and multiple forms of cell death, including apoptosis, ferroptosis, and pyroptosis. These cellular changes ultimately converge on the core pathology of OA: an imbalance between cartilage degeneration and repair.

## 3 The response mechanism of chondrocytes to abnormal stress: the cellular “Translation system”

Chondrocytes respond to abnormal mechanical stress through a sophisticated cascade involving mechanosensors, intracellular signaling pathways, and cellular outcomes. This process begins with mechanical signal perception via ion channels, cytoskeletal remodeling, and primary cilia, followed by signal integration through multiple pathways(Fig. 1), ultimately leading to pathological outcomes including cell death, senescence, and ECM degradation(Table 2).

**Fig. 1:**
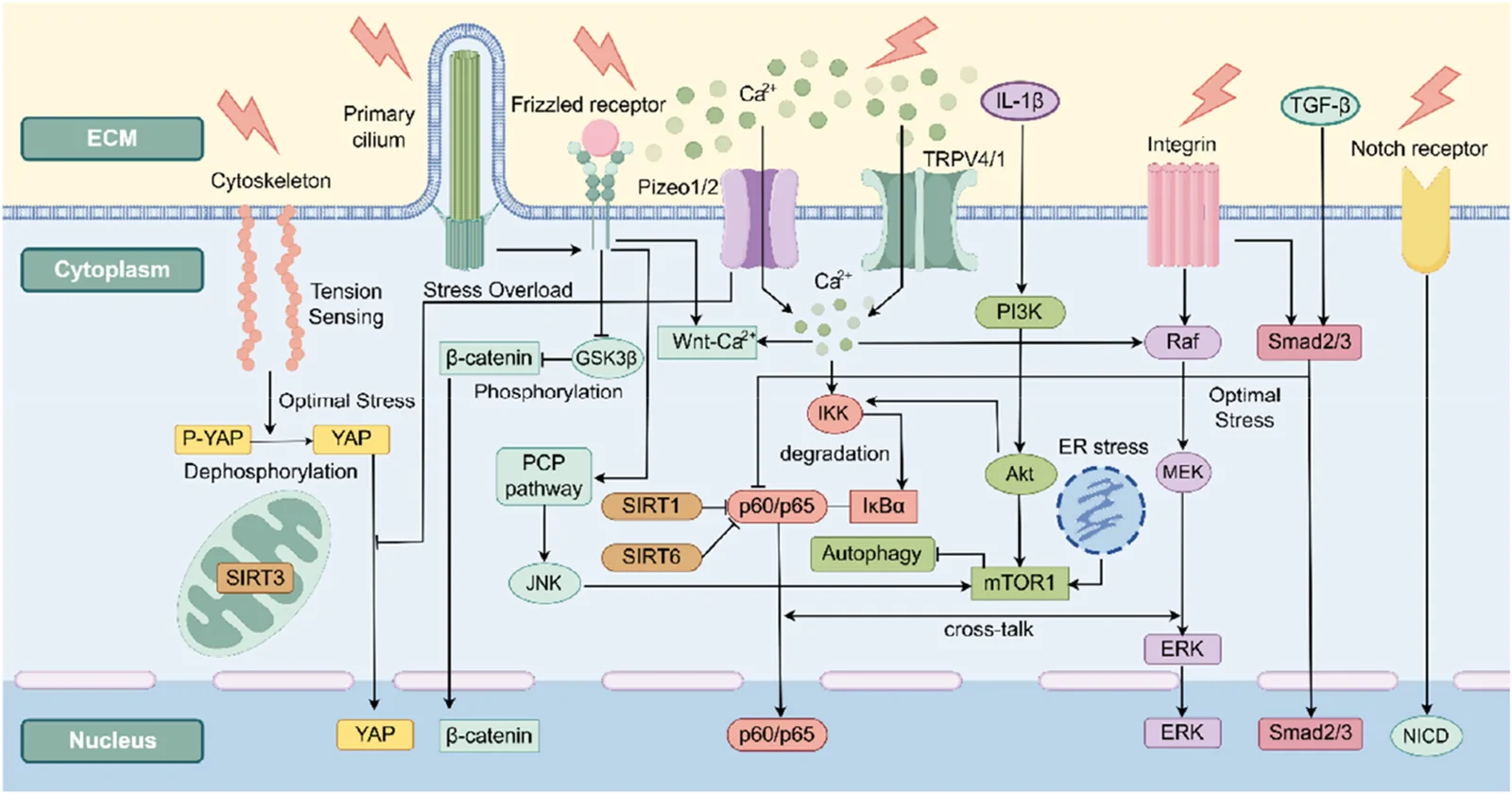
Mechanical signal perception and transduction pathways in chondrocytes in OA.

**Table 2:** Mechanotransductive and regulatory signaling pathways in osteoarthritic chondrocytes.

Pathway	ECM Degradation	Programmed Cell Death	Cellular Senescence	Aberrant Differentiation/Fibrosis	Inflammation/ROS
**NF-κB**	↑ MMPs ↑ ADAMTSs	Apoptosis, pyroptosis, ferroptosis	↑ p16, ↑ p21	NA	↑ IL-1β, IL-6, ↑TNF-α
**Wnt/β-Catenin**	ECM imbalance	NA	NA	Hypertrophic differentiation	NA
**YAP**	↑ MMPs	Apoptosis	↑ p16, ↑ p21 (overload)	NA	NA
**ERK**	Catabolic mediators↑	Apoptosis, autophagy	Dedifferentiation	NA	Inflammation↑
**mTORC1**	Autophagy inhibition	Apoptosis	NA	Hypertrophy inhibition	NA
**TGF-β/Smad2/3**	↑ MMP13, ↑ ADAMTS5, ↓ COL II	Pyroptosis inhibition	Senescence (overload)	Fibrosis	NF-κB suppression
**Notch**	↑ MMPs, ↑ ADAMTSs	NA	NA	NA	↑ IL-8, ↑ IFN-β
**PI3K/Akt**	ECM degradation↑	Apoptosis, pyroptosis	NA	NA	Inflammation↑
**SIRT**	↑ ACAN, ↓ MMPs	Anti-apoptosis, anti-ferroptosis	Anti-senescence	NA	↓ NF-κB, ↓ ROS

### 3.1 Sensors of mechanical signals

Chondrocytes sense mechanical cues through multiple, interconnected structures rather than through a single mechanosensory pathway. Mechanical loading at the joint level is filtered by ECM and PCM, generating local changes in membrane tension, cellular deformation, osmotic conditions, fluid-related cues, and cell-matrix adhesion forces. These cell-scale mechanical states are detected by primary mechanosensors, including mechanosensitive ion channels, integrin-focal adhesion complexes, and primary cilia. Their activation initiates intracellular Ca^2+^ signaling, cytoskeletal remodeling, and downstream signaling networks that regulate chondrocyte homeostasis or pathological responses in OA.

#### 3.1.1 Piezo1 and Piezo2

Piezo1 and Piezo2 are rapidly activated mechanically gated cation channels that respond to membrane tension, cellular deformation, and local mechanical stimuli within milliseconds.^55^ In chondrocytes, Piezo channels are important mediators that convert aberrant mechanical stress into intracellular Ca^2+^ signals. Under 15% tensile stress, sustained or excessive Piezo1 activation can induce Ca^2+^ overload.^56^ In addition to mediating mechanically induced Ca^2+^ influx, Piezo1 has been reported to regulate IFT88 expression, suggesting that Piezo1-dependent mechanosignaling may influence primary ciliary integrity.^57^

#### 3.1.2 TRPV4

Transient receptor potential vanilloid 4 (TRPV4) is another key mechanosensitive cation channel in chondrocytes. TRPV4 can respond to mechanical deformation, osmotic changes, and fluid-related mechanical cues, thereby regulating intracellular Ca^2+^ signaling and chondrocyte homeostasis^58^, ^59^, ^60^. Unlike a purely pathological signaling molecule, TRPV4 appears to have context-dependent functions in cartilage. Under physiological dynamic loading, appropriately regulated TRPV4-mediated Ca^2+^ signaling may contribute to chondrocyte adaptation and matrix homeostasis. In contrast, aberrant mechanical stimulation (e.g., intermittent 2-mm sinusoidal compressive displacements at 0.67 Hz for three 2-h intervals daily) may induce dysregulated TRPV4 activation and pathological Ca^2+^ signaling.^61^ TRPV4 has also been localized to primary cilia, and mechanical stress may enhance its ciliary localization. This process has been associated with HDAC6-dependent modification of soluble tubulin and alterations in ciliary length, suggesting a potential link between TRPV4-mediated mechanosignaling and ciliary structural regulation.^62^

Recent transcriptomic analysis indicates that TRPV4 and Piezo1 activation elicit largely distinct regulatory programs in chondrocytes.^63^ Under supraphysiological deformation, Piezo1 activation downregulated the G2/M checkpoint- and DNA repair-associated genes *PLK1*, *AURKB*, *CDC20*, and *GEN1*, suggesting a potential Piezo1–cell-cycle arrest/DNA repair impairment–cell death axis; in contrast, sustained TRPV4 activation showed substantial overlap with IL-1-induced gene suppression.

#### 3.1.3 Primary cilia

Primary cilia are solitary, microtubule-based organelles that project from the surface of chondrocytes and function as important mechanosensory and signaling compartments. Early electron microscopy studies showed that primary cilia can align with the surrounding ECM, providing initial evidence that they respond to mechanical forces.^64^ Mechanical stimuli, including external loading, substrate stiffness, and fluid shear stress (FSS), can induce bending or deflection of primary cilia.^65^, ^66^, ^67^ These structural changes are thought to be detected by mechanosensitive ion channels or other signaling proteins located in the ciliary membrane or at the ciliary base. The resulting changes in ion flux, particularly intracellular Ca^2+^ signaling, convert mechanical inputs into biochemical signals that can be transmitted to the cytoplasm and nucleus.^68^^,^^69^

#### 3.1.4 Integrin-focal adhesion complexes

Integrins are heterodimeric transmembrane receptors composed of α- and β-subunits that connect chondrocytes to ECM components such as collagen and fibronectin. Their extracellular domains bind matrix ligands, whereas their intracellular domains associate with adaptor and signaling proteins to connect the ECM with the actin cytoskeleton. Because integrins lack intrinsic enzymatic activity in their cytoplasmic domains, they rely on associated proteins to assemble signaling complexes at the cytoplasmic face of the plasma membrane.^70^, ^71^, ^72^ This organization enables integrins to function as mechanosensitive interfaces that detect changes in cell–matrix adhesion and force transmission.^73^

Changes in ECM stiffness, fibrillar organization, or ligand presentation can alter the traction forces generated by chondrocytes and the mechanical stability of integrin–ECM bonds. These force-dependent changes may promote the transition of integrins from a bent, low-affinity conformation toward an extended, higher-affinity state, followed by integrin clustering and focal adhesion maturation.^74^, ^75^, ^76^, ^77^ Within these adhesion complexes, talin and kindlin regulate integrin activation, whereas talin, vinculin, paxillin, and other adaptor proteins connect integrins to actin filaments and organize signaling components at the cytoplasmic face of the adhesion site.^78^, ^79^, ^80^, ^81^, ^82^, ^83^, ^84^ Mechanical tension can induce conformational or post-translational changes in focal adhesion proteins, including talin, vinculin, paxillin, and p130Cas, while promoting the phosphorylation and activation of focal adhesion kinase (FAK), Src, and associated adhesion proteins.^77^^,^^85^

The resulting integrin–focal adhesion signals regulate RhoA/ROCK-dependent actomyosin contractility, cytoskeletal remodeling, and the transmission of mechanical tension toward the nucleus.^86^ These responses can further influence MAPK, NF-κB, PI3K/Akt, and YAP/TAZ signaling, thereby modulating chondrocyte spreading, cytoskeletal organization, matrix synthesis, inflammatory activation, and the expression of matrix-degrading enzymes such as MMPs and ADAMTS.^87^ In OA, pathological changes in ECM and PCM properties may therefore disrupt integrin-mediated force sensing and focal adhesion signaling, contributing to abnormal chondrocyte mechanotransduction and cartilage degeneration.^85^^,^^86^^,^^88^

### 3.2 Intracellular signal integration following mechanosensing

Following activation of primary mechanosensors, Ca^2+^ serves as a major early intracellular signal that links mechanical inputs to downstream signaling networks in chondrocytes. The amplitude, duration, and subcellular distribution of mechanically induced Ca^2+^ signals may determine whether chondrocytes undergo adaptive responses or pathological signaling under excessive or persistent loading. Ca^2+^ dysregulation has been implicated in the activation of MAPK and NF-κB signaling and may also modulate Wnt/β-catenin signaling, cytoskeletal remodeling, mitochondrial function, and inflammatory responses.^89^, ^90^, ^91^, ^92^, ^93^ Thus, Ca^2+^ acts as a signaling hub through which inputs from distinct mechanosensory structures are integrated before being relayed to downstream biochemical, cytoskeletal, and transcriptional pathways.

#### 3.2.1 FAK/Src and RhoA/ROCK-mediated cytoskeletal integration

Following force-dependent integrin activation and focal adhesion maturation, FAK is recruited to adhesion complexes and undergoes autophosphorylation, creating docking sites for Src and other signaling molecules. The formation of the FAK/Src complex promotes phosphorylation of focal adhesion-associated proteins and amplifies signals initiated at the ECM-integrin interface.^77^^,^^85^ In this manner, FAK/Src signaling translates changes in ECM stiffness, ligand presentation, and cell-matrix force transmission into intracellular biochemical responses.

A major downstream consequence of FAK/Src activation is the regulation of Rho family GTPases, particularly RhoA, and its effector Rho-associated coiled-coil-containing protein kinase (ROCK). RhoA/ROCK signaling promotes actomyosin contractility by enhancing myosin light-chain phosphorylation and inhibiting myosin phosphatase activity, thereby increasing actin stress-fiber formation and cellular tension. This process also promotes focal adhesion maturation, reinforcing the physical linkage among integrins, focal adhesion proteins, and the actin cytoskeleton. Accordingly, FAK/Src and RhoA/ROCK form a coupled signaling module that integrates extracellular mechanical information with cytoskeletal architecture and intracellular force generation.^94^

In chondrocytes, cytoskeletal remodeling alters cell shape, mechanical sensitivity, and the transmission of force toward the nucleus. The FAK/Src-RhoA/ROCK-actomyosin axis may further regulate MAPK signaling and YAP nuclear localization, thereby linking matrix-derived mechanical cues to inflammatory, metabolic, and transcriptional responses.^91^ Under physiological loading, this signaling axis may facilitate cellular adaptation to the mechanical environment. However, persistent ECM remodeling or excessive mechanical stress may result in aberrant focal adhesion signaling and cytoskeletal tension, contributing to altered matrix metabolism and OA-associated chondrocyte dysfunction.

#### 3.2.2 MAPK signaling

Mitogen-activated protein kinase (MAPK) signaling functions as an important intracellular pathway downstream of mechanical stimulation in chondrocytes. The MAPK family includes several major branches, among which ERK, JNK, and p38 regulate cellular responses to mechanical stress, inflammation, and changes in the extracellular environment.^95^ These pathways can integrate signals originating from mechanosensitive ion channels, integrin-associated focal adhesions, and cytoskeletal remodeling, thereby linking early mechanical sensing to changes in chondrocyte phenotype.

Mechanical stimulation can activate the classical Raf–MEK–ERK cascade. In this process, mechanically induced Ca^2+^ influx activates Raf, which phosphorylates MEK; MEK then phosphorylates ERK at the conserved TEY motif, resulting in ERK activation and its translocation into the nucleus.^93^ Integrin-mediated signaling also contributes to ERK activation. Excessive mechanical stress (DMM surgery) increases integrin αVβ3 expression and promotes the phosphorylation of FAK and ERK, linking abnormal ECM–cell interactions to downstream MAPK signaling.^91^ These findings suggest that Ca^2+^-dependent and integrin–FAK-dependent mechanisms may provide complementary routes for transmitting mechanical information to the ERK pathway.

MAPK activation is strongly dependent on the type, intensity, and duration of mechanical stimulation. Low-intensity fluid shear stress, defined in one study as FSS ≤ 2 dyn/cm^2^, enhanced ERK5 activity through inhibition of miR-143-3p and protected chondrocytes from inflammatory injury.^96^^,^^97^ ERK5 activation also increased KLF4 expression, which was associated with reduced IL-1β-induced apoptosis and enhanced the expression of cartilage-related matrix components, including type II collagen and aggrecan.^96^^,^^97^ In contrast, excessive mechanical stress-associated ERK activation promoted inflammatory and catabolic mediator expression.^91^ These observations indicate that MAPK signaling is not uniformly pathogenic; rather, its biological effects depend on the mechanical context and the specific MAPK branch that is activated.

The cellular environment further influences MAPK-dependent responses. In two-dimensional culture, abnormal nuclear mechanics and stress-induced mitochondrial damage activate ERK1/2 and p38 MAPK signaling, which is associated with chondrocyte dedifferentiation and reduced cartilage-specific ECM production.^98^ In addition, ERK, JNK, and NF-κB signaling can act cooperatively in mediating chondrocyte responses to mechanical stimulation.^99^

#### 3.2.3 NF-κB as an inflammatory signaling hub

Nuclear factor-kappa B (NF-kB) is a conserved family of transcription factors that regulates inflammatory, catabolic, and stress-responsive gene expression in chondrocytes.^100^ Under basal conditions, NF-kB is retained in the cytoplasm through its association with inhibitor of kappa B (IkB) proteins, particularly IkBa. Upon activation, NF-kB, mainly the p65-containing complex, translocates to the nucleus and initiates transcriptional programs that regulate inflammation, extracellular matrix metabolism, cellular senescence, and cell death.

Following primary mechanosensor activation, Ca^2+^ signaling provides an important route through which abnormal mechanical stimuli can promote NF-kB activation. TRPV4-mediated signaling has been reported to activate NF-kB through Ca^2+^dependent mechanisms and the HMGB1/TLR4 axis.^90^ Piezo1-mediated Ca^2+^ influx may similarly link mechanical overload to inflammatory signaling. In OA chondrocytes, aberrant mechanical stress (sinusoidal stretching at 15% strain and 0.5 Hz for 12 h) may activate Piezo1-mediated Ca^2+^ influx and induce mitochondrial Ca^2+^ overload, mitochondrial dysfunction, and cytosolic release of mitochondrial DNA (mtDNA).^101^ Cytosolic mtDNA can activate the cGAS-STING pathway, which may promote inflammatory signaling, cellular senescence, and cartilage catabolism. This Piezo1-mitochondrial stress-mtDNA/cGAS-STING axis provides a potential mechanistic link between pathological mechanical stress and persistent inflammatory degeneration in OA.^102^

In mechanically stretched nucleus pulposus cells, Piezo1 activation increased intracellular Ca^2+^ levels, activated NF-kB, and promoted NLRP3 and pro-IL-1β expression; the elevated Ca^2+^ signal also contributed to NLRP3 inflammasome assembly, caspase-1 activation, and IL-1β secretion.^103^ Piezo1 silencing or inhibition of the Ca^2+^/NF-kB pathway attenuated NLRP3 inflammasome activation in this model.^103^ Although this mechanism has not yet been fully established in chondrocytes, it provides a plausible mechanistic model through which pathological mechanical stress may couple Piezo1 activation to inflammatory responses in OA cartilage.

In addition to ion-channel activation, pathological cyclic tensile stress, compressive stress, and high-intensity shear stress may activate NF-κB in chondrocytes through integrin- and focal adhesion-associated signaling, inflammatory signaling, and ROS generation, as reflected by p65 phosphorylation and nuclear translocation.^96^^,^^104^, ^105^, ^106^, ^107^, ^108^ These mechanical and biochemical inputs may converge on the canonical IkB kinase (IKK)-IkBa pathway. Upon activation, the IKK complex phosphorylates IkBa and promotes its degradation, releasing NF-kB from cytoplasmic sequestration. Mechanical stimulation may also enhance NF-kB transcriptional activity through direct phosphorylation of the p65 subunit.^109^, ^110^, ^111^

NF-kB functions as an inflammatory signaling hub because its activity is further shaped by inputs from multiple intracellular stress-response pathways. ERK, JNK, and NF-kB signaling jointly mediate chondrocyte responses to mechanical stimulation, indicating functional integration between MAPK and NF-kB pathways.^99^ Oxidative stress can also reinforce NF-kB activation; for example, the CCL4/CCR5 axis mediates ROS-associated inflammatory responses through NF-kB signaling.^112^^,^^113^ Conversely, Nrf2 suppresses NF-kB-associated inflammation while promoting antioxidant responses, and TGF-β1/Smad2/3 signaling protects chondrocytes from pyroptosis partly through NF-kB inhibition under physiological mechanical conditions.^114^, ^115^, ^116^ SIRT1, SIRT3, and SIRT6 may further limit NF-kB activity through epigenetic regulation, autophagy, mitochondrial quality control, and reduction of oxidative stress.^117^^,^^118^

Sustained NF-kB activation promotes pathological chondrocyte responses in OA. It induces pro-inflammatory cytokines and mediators, including IL-1β, IL-6, TNF-alpha, COX-2, and iNOS, thereby amplifying the inflammatory microenvironment.^119^, ^120^, ^121^ NF-kB also promotes extracellular matrix degradation by upregulating MMPs and ADAMTSs, which mediate the breakdown of type II collagen and proteoglycans.^110^^,^^122^^,^^123^ In addition, persistent NF-kB activity is associated with apoptosis, pyroptosis, ferroptosis, and senescence-associated increases in p16 and p21 expression.^21^^,^^89^^,^^124^, ^125^, ^126^, ^127^ Collectively, NF-kB integrates mechanically induced Ca^2+^ signaling with MAPK-, ROS-, and inflammation-associated inputs, translating persistent abnormal mechanical stress into inflammation, matrix catabolism, and chondrocyte dysfunction.

#### 3.2.4 Wnt/β-catenin signaling

Wnt signaling is an important regulator of chondrocyte differentiation, matrix metabolism, and OA progression. It includes canonical β-catenin-dependent signaling and non-canonical β-catenin-independent pathways.^128^ In the canonical pathway, Wnt ligands bind to Frizzled receptors and low-density lipoprotein receptor-related protein 5/6 (LRP5/6) co-receptors. This inhibits the β-catenin destruction complex, preventing glycogen synthase kinase-3 (GSK-3)-dependent β-catenin phosphorylation and degradation. Stabilized β-catenin subsequently accumulates in the cytoplasm, translocates to the nucleus, and interacts with T-cell factor/lymphoid enhancer-binding factor (TCF/LEF) transcription factors to regulate target-gene expression.^92^^,^^129^

Mechanical inputs may influence canonical Wnt/β-catenin signaling through multiple mechanosensory and intracellular signaling routes. Mechanically induced Ca^2+^ signaling downstream of Piezo1 and TRPV4 has been implicated in the enhancement of canonical Wnt/β-catenin activity.^91^^,^^92^ In addition, primary cilia and integrin-associated signaling have been implicated in Wnt regulation in chondrocytes. However, the molecular steps connecting individual mechanical stimuli or mechanosensors to β-catenin stabilization remain incompletely defined. Wnt/β-catenin should therefore be considered a downstream signaling node responsive to convergent mechanical inputs rather than a primary mechanosensor.

Non-canonical Wnt pathways may provide additional links between mechanical cues and cytoskeletal or Ca^2+^dependent responses. Wnt/planar cell polarity signaling activates Rho GTPases and JNK through Frizzled receptors, thereby regulating cell polarity and migration.^130^^,^^131^ The Wnt/Ca^2+^ pathway induces intracellular Ca^2+^ release through phospholipase C and subsequently activates CaMKII and PKC, which can influence cell morphology.^132^ Under mechanical stress, these non-canonical pathways may modulate chondrocyte alignment and Ca^2+^ homeostasis.^133^ Thus, Wnt signaling may both receive mechanically induced Ca^2+^ signals and, through its non-canonical branches, further regulate Ca^2+^ and cytoskeleton-associated responses.

Dysregulated Wnt/β-catenin signaling is associated with pathological chondrocyte phenotypes in OA. Excessive β-catenin activity can promote hypertrophic differentiation and disturb the balance between matrix synthesis and degradation.^92^^,^^128^ Given its integration with Ca^2+^ signaling, cytoskeletal regulation, and JNK activity, abnormal Wnt signaling may contribute to the loss of stable chondrocyte phenotype and progressive cartilage degeneration. Nevertheless, the load-dependent effects of Wnt signaling and the direct contribution of individual mechanosensors require further clarification in OA chondrocytes.

### 3.3 Transcriptional regulatory layer of mechanotransduction

#### 3.3.1 YAP-mediated mechanosensitive transcription

Yes-associated protein (YAP) is a mechanosensitive transcriptional co-activator that links intracellular mechanical states to chondrocyte gene expression and cell fate 103. Rather than functioning as a primary mechanosensor, YAP acts downstream of cytoskeletal remodeling, focal adhesion signaling, and mechanical stress-responsive pathways. Its activity is regulated mainly by phosphorylation and subcellular localization. Nuclear YAP interacts with TEAD transcription factors and other transcriptional regulators, whereas cytoplasmic retention limits its transcriptional activity and may promote its degradation.^134^

The transcriptional response mediated by YAP is dependent on the intensity and context of mechanical stimulation. Moderate mechanical stress can promote cytoskeletal and adhesion-complex remodeling and enhance YAP activation through RhoA/ROCK- and p38 MAPK-associated mechanisms.^134^^,^^135^ Activated YAP may then regulate gene expression related to chondrocyte survival, matrix metabolism, and cellular adaptation. In contrast, stress under cyclic equibiaxial stretching at 0.3 Hz and 3% elongation for 9 h can activate Piezo1-associated signaling and impair YAP nuclear translocation, a response that is associated with increased expression of senescence markers, including p16 and p21, and reduced ECM synthetic capacity.^10^^,^^136^^,^^137^

Dysregulated YAP activity is associated with pathological changes in chondrocyte phenotype. YAP has been linked to increased expression of matrix metalloproteinases, pro-inflammatory mediators, and apoptotic factors, thereby contributing to ECM degradation and cartilage damage.^137^, ^138^, ^139^ Abnormal YAP responses to changes in matrix viscoelasticity have also been associated with impaired mechanosensing in OA chondrocytes.^137^^,^^140^

#### 3.3.2 NF-κB-dependent inflammatory and catabolic transcription

NF-κB is a central transcriptional regulator that connects mechanically induced intracellular signaling with inflammatory and catabolic gene expression. Following activation and nuclear translocation of the p65-containing NF-κB complex, NF-κB binds regulatory regions of target genes and initiates transcriptional programs involved in inflammation, matrix degradation, cellular senescence, and cell death.^100^^,^^109^, ^110^, ^111^

In chondrocytes under cyclic compression of 0–40 kPa at 1 Hz for 9 h, NF-κB-dependent transcription increases the expression of inflammatory cytokines and mediators, including IL-1β, IL-6, TNF-α, COX-2, and iNOS.^119^, ^120^, ^121^ These factors can amplify inflammatory signaling within the joint and further increase the responsiveness of chondrocytes to mechanical and biochemical stress. NF-κB also promotes the expression of MMPs and ADAMTSs, thereby contributing to the degradation of type II collagen and proteoglycans.^110^^,^^122^^,^^123^

Persistent NF-κB-dependent transcription is additionally associated with pathological cell-state transitions. It has been linked to apoptosis, pyroptosis, and ferroptosis, as well as increased expression of senescence-associated regulators such as p16 and p21.^21^^,^^89^^,^^124^, ^125^, ^126^, ^127^ Therefore, NF-κB serves as a transcriptional convergence point at which mechanically induced Ca^2+^ signaling and other stress-responsive pathways are translated into inflammatory amplification, matrix catabolism, senescence, and chondrocyte death.

#### 3.3.3 Wnt/β-catenin-associated transcriptional regulation

The Wnt/β-catenin pathway regulates transcriptional programs involved in chondrocyte differentiation, hypertrophic change, and ECM metabolism.^128^ In the canonical pathway, Wnt binding to Frizzled and LRP5/6 inhibits β-catenin degradation. Stabilized β-catenin accumulates in the cytoplasm and translocates to the nucleus, where it interacts with TCF/LEF transcription factors to regulate the expression of Wnt-responsive genes.^92^^,^^129^

Mechanical inputs may influence β-catenin-dependent transcription through several upstream signaling routes. Mechanically induced Ca^2+^ signaling downstream of Piezo1 and TRPV4 has been implicated in the regulation of canonical Wnt/β-catenin activity.^91^^,^^92^ Mechanical stress may also reduce the expression of secreted Frizzled-related proteins, thereby weakening extracellular inhibition of Wnt signaling and favoring β-catenin accumulation.^92^ In addition, primary cilia are involved in the regulation of Wnt-related signaling in chondrocytes, although the specific molecular connection between ciliary mechanosensation and β-catenin-dependent transcription remains incompletely defined.^141^, ^142^, ^143^

Wnt signaling also regulates transcription indirectly through its non-canonical branches. Wnt/planar cell polarity signaling can activate Rho GTPases and JNK, thereby influencing cell polarity, migration, and cytoskeletal organization.^130^^,^^131^ The Wnt/Ca^2+^ pathway can activate CaMKII and PKC following phospholipase C-dependent Ca^2+^ release, providing an additional route through which Wnt signaling may influence cell morphology and cellular responses to mechanical stress.^132^^,^^133^ Dysregulated canonical and non-canonical Wnt signaling may therefore alter chondrocyte differentiation, promote hypertrophic changes, and disturb the balance between matrix synthesis and degradation during OA progression.^92^^,^^128^

#### 3.3.4 MAPK-associated transcriptional programs

MAPK pathways regulate gene expression by transmitting mechanical, inflammatory, and metabolic signals to nuclear transcriptional programs. The major MAPK branches, including ERK, JNK, and p38, can produce distinct but overlapping transcriptional responses depending on the type, intensity, and duration of the stimulus.^95^ In this context, MAPK pathways should be regarded not only as cytoplasmic signaling cascades but also as regulators of stress-responsive gene expression in mechanically stimulated chondrocytes.

ERK activation can promote nuclear signaling and alter the expression of genes involved in proliferation, inflammation, matrix metabolism, and chondrocyte phenotype. Under Low fluid shear stress (FSS, ≤ 2 dyn/cm^2^), ERK5 activation through inhibition of miR-143-3p increases KLF4 expression and is associated with reduced IL-1β-induced apoptosis and enhanced expression of cartilage-related matrix components, including type II collagen and aggrecan.^96^^,^^97^ By contrast, excessive mechanical stress-associated ERK activation (under DMM surgery) promotes inflammatory and catabolic mediator expression.^91^ In two-dimensional culture, abnormal nuclear mechanics and stress-associated mitochondrial damage have also been linked to ERK1/2- and p38-dependent changes in chondrocyte phenotype, including dedifferentiation and reduced cartilage-specific ECM production.^98^

MAPK-associated transcriptional responses interact with other regulatory modules. ERK, JNK, and NF-κB jointly mediate chondrocyte responses to mechanical stimulation, allowing mechanical stress to influence inflammatory and catabolic gene expression through coordinated signaling.^99^ MAPK pathways may also interact with YAP and Wnt/β-catenin signaling, although the direction and functional significance of these interactions may vary according to the mechanical and inflammatory context. Collectively, MAPK signaling contributes to the transcriptional interpretation of mechanical stress by regulating gene programs related to inflammation, matrix metabolism, apoptosis, and maintenance or loss of the differentiated chondrocyte phenotype.^91^^,^^97^, ^98^, ^99^

#### 3.3.5 Convergence of mechanotransduction-responsive transcriptional programs

YAP, NF-κB, β-catenin, and MAPK-associated transcriptional programs represent interconnected downstream responses rather than independent mechanical sensors. Mechanically induced Ca^2+^ signaling, integrin-associated signaling, cytoskeletal remodeling, oxidative stress, and inflammatory mediators can influence these transcriptional regulators at different stages of the response. Their combined activity determines whether chondrocytes activate adaptive programs that support matrix homeostasis or pathological programs associated with inflammation, ECM degradation, senescence, aberrant differentiation, and cell death.

The functional outcome is therefore determined by the integration of pathway activity rather than by activation of a single transcriptional regulator. For example, regulated YAP and ERK5 activity may support cellular adaptation and matrix maintenance under appropriate mechanical conditions, whereas persistent NF-κB activation, excessive Wnt/β-catenin activity, or dysregulated MAPK signaling may promote catabolism and loss of the chondrocyte phenotype.^91^^,^^96^^,^^97^^,^^137^^,^^138^ This transcriptional layer provides the final intracellular step through which mechanically induced signals are converted into stable changes in chondrocyte behavior and cartilage homeostasis.

### 3.4 Downstream biological consequences of abnormal mechanotransduction

The diverse mechanotransduction pathways activated in OA chondrocytes ultimately converge on a limited number of biological outcomes. Rather than acting in isolation, Ca^2+^ signaling, FAK/Src and cytoskeletal pathways, MAPK, NF-kB, Wnt/β-catenin, YAP, PI3K/Akt-mTOR, and oxidative stress-related mechanisms collectively determine whether chondrocytes maintain matrix homeostasis or shift toward a degenerative phenotype. These downstream consequences include matrix imbalance, inflammatory amplification, pathological changes in chondrocyte phenotype, and progressive OA development.

#### 3.4.1 Imbalance between matrix synthesis and degradation

One of the most important consequences of abnormal mechanical signaling is disruption of the balance between extracellular matrix synthesis and degradation. Under pathological mechanical conditions, anabolic gene expression is reduced, including decreased synthesis of cartilage-specific matrix components such as type II collagen and aggrecan, whereas catabolic gene expression is enhanced.^137^^,^^138^^,^^144^ In parallel, multiple mechanotransduction-responsive pathways, particularly NF-kB, ERK-associated signaling, YAP dysregulation, and Wnt/β-catenin-related responses, increase the expression of matrix-degrading enzymes, including MMPs and ADAMTSs.^91^^,^^110^^,^^122^^,^^123^^,^^137^^,^^138^

This imbalance weakens both the ECM and the pericellular matrix, thereby altering the mechanical environment surrounding chondrocytes. As matrix integrity declines, force transmission, osmotic buffering, and cell-matrix interactions become increasingly abnormal, which further perturbs mechanosensing and reinforces downstream catabolic signaling. Thus, matrix degradation is not only a consequence of abnormal mechanotransduction but also a driver of its further amplification.

#### 3.4.2 Amplification of inflammatory responses

Abnormal mechanical stress also promotes a persistent inflammatory state in chondrocytes and surrounding joint tissues. NF-kB-dependent transcription induces inflammatory mediators including IL-1β, IL-6, TNF-alpha, COX-2, and iNOS, while MAPK-associated signaling can further enhance inflammatory and stress-responsive gene expression.^91^^,^^99^^,^^119^, ^120^, ^121^ Mechanosensitive ion-channel signaling, oxidative stress, and inflammasome-related pathways may additionally contribute to this inflammatory amplification.^89^^,^^90^^,^^112^^,^^113^^,^^138^

Once established, inflammation feeds back on mechanotransduction itself. Pro-inflammatory cytokines and oxidative stress can alter ion-channel activity, adhesion-associated signaling, mitochondrial function, and cytoskeletal organization, thereby changing how chondrocytes interpret subsequent mechanical stimuli.^89^^,^^90^^,^^138^^,^^145^ In this way, inflammation is both an output of abnormal mechanotransduction and a secondary factor that sustains or worsens signaling dysregulation.

#### 3.4.3 Pathological changes in chondrocyte phenotype

Persistent activation or dysregulation of mechanotransduction pathways also alters chondrocyte phenotype. Several downstream pathways, including YAP, Wnt/β-catenin, ERK1/2, p38, and NF-kB, have been associated with hypertrophic differentiation, dedifferentiation, senescence, and programmed cell death.^21^^,^^89^^,^^92^^,^^98^^,^^124^, ^125^, ^126^, ^127^^,^^137^^,^^138^ These changes are accompanied by reduced matrix-producing capacity, loss of the stable articular chondrocyte phenotype, and impaired ability to maintain tissue homeostasis.

Mechanical overload-induced YAP dysregulation has been linked to increased expression of senescence markers such as p16 and p21 and reduced ECM synthesis.^8^^,^^106^ Excessive NF-kB activation has similarly been associated with apoptosis, pyroptosis, ferroptosis, and senescence-related responses.^21^^,^^89^^,^^124^, ^125^, ^126^, ^127^ In parallel, abnormal Wnt/β-catenin signaling may promote hypertrophic differentiation, whereas ERK1/2- and p38-associated signaling has been linked to dedifferentiation and reduced cartilage-specific ECM production.^92^^,^^98^ Collectively, these phenotypic alterations diminish the capacity of chondrocytes to repair or adapt to mechanical stress.

#### 3.4.4 Self-reinforcing progression of OA

The overall consequence of these processes is the establishment of a self-reinforcing pathological cycle in OA. Mechanical abnormalities, matrix degradation, inflammatory signaling, oxidative stress, and altered cell phenotype do not act as separate events; instead, they mutually amplify one another. Changes in ECM and PCM structure modify the mechanical signals reaching chondrocytes, abnormal mechanotransduction activates catabolic and inflammatory pathways, and these pathways further degrade the matrix and impair cell function.^91^^,^^137^^,^^138^^,^^144^

OA progression can therefore be understood as a vicious cycle in which matrix alteration leads to abnormal mechanical signaling, abnormal signaling drives chondrocyte dysfunction, and chondrocyte dysfunction accelerates further matrix destruction and inflammatory activation. This integrated view emphasizes that the pathological significance of mechanotransduction lies not in the activation of any single pathway, but in the cumulative shift of chondrocytes from adaptive homeostasis toward chronic degeneration.

### 3.5 Crosstalk and feedback regulation among mechanotransduction pathways

Mechanotransduction in chondrocytes is mediated by interconnected signaling pathways rather than by independent linear cascades. Signals generated by mechanosensitive ion channels, integrin–focal adhesion complexes, and cytoskeletal remodeling can converge on common intracellular and transcriptional regulators. Piezo1/2- and TRPV4-associated Ca^2+^ signaling has been implicated in the regulation of MAPK and NF-κB activity, as well as in downstream inflammatory and catabolic responses.^89^, ^90^, ^91^, ^92^ Ca^2+^-dependent signaling may also interact with cytoskeletal remodeling, although the specific molecular mechanisms linking mechanically induced Ca^2+^ signals to cytoskeletal tension in OA chondrocytes remain incompletely defined.

Integrin-mediated signaling provides another major route for signal integration. Changes in ECM properties and cell–matrix force transmission promote focal adhesion-associated signaling, including FAK/Src activation, which can regulate RhoA/ROCK-dependent actomyosin contractility and cytoskeletal organization.^91^ This mechanical signaling axis may influence downstream MAPK signaling and YAP activity, thereby connecting ECM-derived mechanical information with transcriptional regulation.^91^^,^^134^^,^^135^ In chondrocytes, the effects of these pathways are context dependent: appropriately regulated mechanical signaling may support cellular adaptation and matrix maintenance, whereas excessive or persistent activation may promote inflammatory responses, matrix degradation, senescence, and loss of the differentiated phenotype.^91^^,^^96^^,^^97^^,^^137^^,^^138^

At the transcriptional level, YAP, NF-κB, β-catenin, and MAPK-associated transcriptional programs represent convergent downstream responses to mechanical and biochemical stimuli. NF-κB primarily promotes inflammatory and catabolic gene expression, whereas YAP and β-catenin regulate cell-state transitions, matrix metabolism, and chondrocyte differentiation.^92^^,^^100^^,^^128^^,^^134^^,^^137^^,^^138^ ERK, JNK, and p38 can further modify stress-responsive transcriptional programs and cooperate with NF-κB signaling during mechanical and inflammatory stimulation.^95^^,^^99^ These regulators should therefore be viewed as interconnected transcriptional modules that collectively determine the balance between adaptive homeostasis and pathological chondrocyte responses, rather than as independent mechanosensory pathways.

Feedback regulation further amplifies the effects of abnormal mechanical stress. Mechanical stress can initiate ion-channel, focal adhesion, MAPK, NF-κB, and oxidative stress responses, which promote the production of inflammatory mediators and matrix-degrading enzymes.^112^^,^^113^^,^^146^ Subsequent degradation of the ECM and PCM may alter matrix stiffness, ligand presentation, cell–matrix adhesion, and the local mechanical environment, thereby changing the mechanical signals perceived by chondrocytes. Inflammatory mediators and matrix-derived signals may also further enhance catabolic and stress-responsive signaling. This creates a self-reinforcing cycle in which matrix alteration promotes abnormal mechanotransduction, abnormal mechanotransduction drives chondrocyte dysfunction, and chondrocyte dysfunction accelerates additional matrix degradation and inflammation.

Overall, the mechanotransduction network can be summarized as a multilayered system: Piezo1/2 and TRPV4 generate early Ca^2+^-dependent signals; integrin–FAK/Src signaling regulates cytoskeletal tension and downstream signaling; MAPK and NF-κB integrate stress and inflammatory inputs; and YAP and β-catenin contribute to transcriptional regulation of chondrocyte phenotype and matrix metabolism. The combined activity of these pathways, rather than the activation of any individual component, determines the cellular response to mechanical stress.

Under physiological conditions, transient and appropriately regulated signaling may promote chondrocyte adaptation and matrix homeostasis. Under pathological conditions, sustained mechanical stress, ECM/PCM degradation, inflammation, and oxidative stress disrupt this balance and establish a positive-feedback loop that favors catabolism, senescence, cell death, and OA progression.

## 4 Treatment approaches targeting abnormal mechanical environments in chondrocytes

Deeper understanding of cascading reactions from macroscopic loading to cellular signaling in OA has opened new multi-layered, multi-targeted treatment pathways. These strategies no longer focus solely on symptomatic treatment but actively intervene at various stages of mechanotransduction, achieving a transition from theoretical “mechanobiology” to clinical “mechanomedicine.” Currently, these interventions can be categorized into three levels: reshaping the macromechanical environment, microregulating cellular signaling networks, and utilizing advanced biomaterials to simulate and repair healthy microenvironments.

### 4.1 Macromechanical control: reshaping joint mechanical balance

Among all interventions, directly modulating the joint macromechanical environment is the most fundamental and logical strategy. For most patients in early-to-mid-stage disease, physical therapy and exercise interventions effectively slow OA progression by modulating the mechanical environment.^20^^,^^21^^,^^124^^,^^147^ The dose-response relationship of mechanical loading is particularly noteworthy: early mechanical unloading (i.e., rest) following joint injury positively affects OA progression, but this benefit may be difficult to sustain in later stages.^148^^,^^149^ In mid-to-late-stage disease, moderate mechanical loading demonstrates clear protective effects, with one key mechanism being regulation of synovial macrophage M1/M2 polarization, thereby systematically reducing synovial inflammation.^22^ Specific exercise modalities exhibit distinct biological effects. For example, moderate-intensity treadmill exercise reduces inflammation and excessive autophagy by downregulating mitochondrial fusion protein 2 (MFN2), while swimming and novel “KOA Pendulum Therapy” (KOAPT) improve patient symptoms through different mechanisms.^150^, ^151^, ^152^ In addition to exercise, certain physical therapies, such as mineral-rich hot spring baths, show potential for joint tissue protection due to their anti-inflammatory and antioxidant properties.^153^ For end-stage OA, surgical interventions (such as osteotomy and joint replacement) remain the definitive means of restoring mechanical balance.^154^, ^155^, ^156^, ^157^, ^158^, ^159^, ^160^, ^161^, ^162^ The success of these macroscopic interventions underscores the central role of mechanical environment in OA pathology and guides development of more precise microscopic intervention strategies (Fig. 2).

**Fig. 2:**
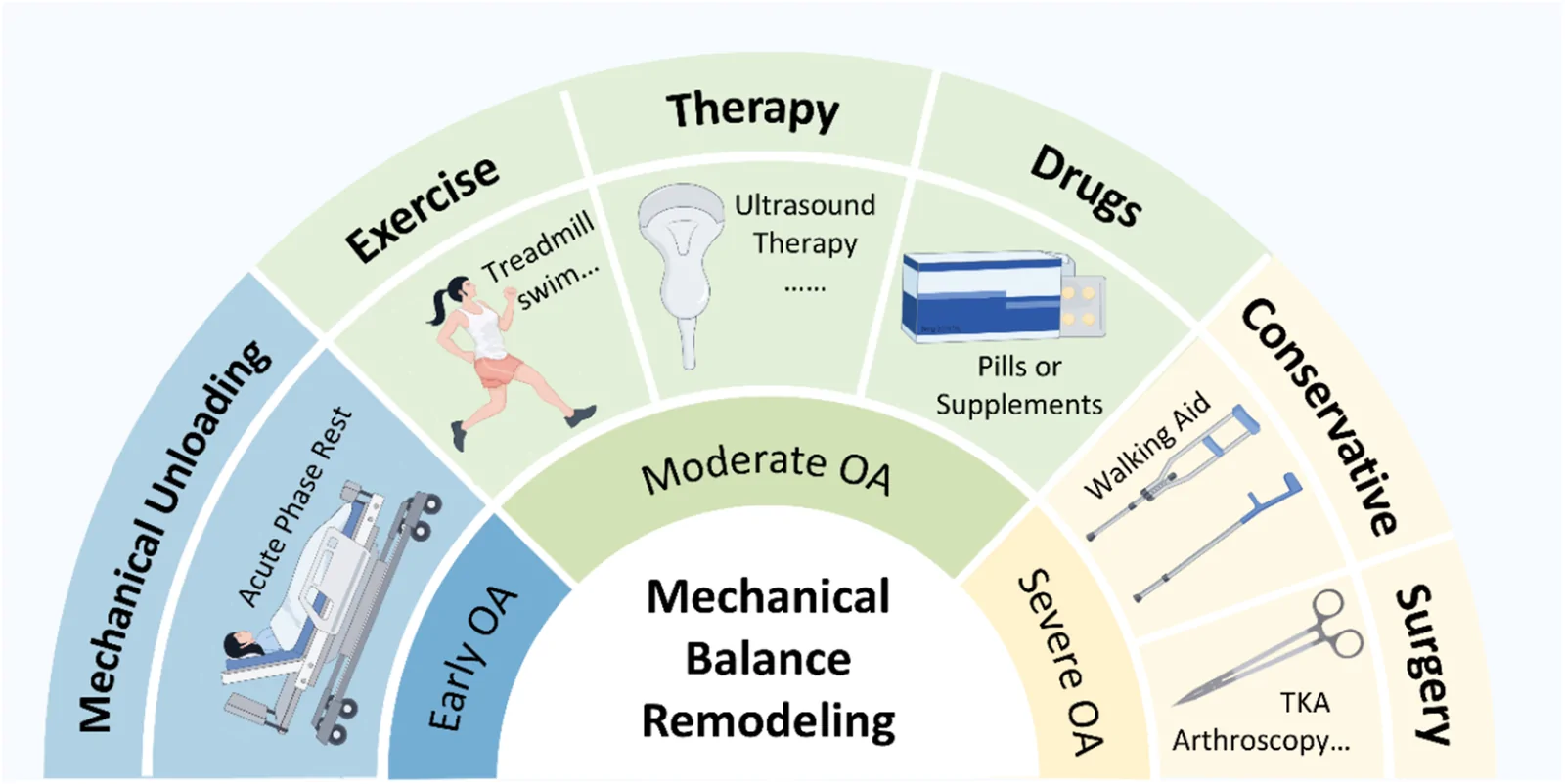
Strategies for reshaping the macromechanical environment of OA.

### 4.2 Microregulation: Targeting the cellular signal transduction system

#### 4.2.1 Precise blockade: targeting key signaling pathways and mechanoreceptors

Other intervention strategies involve precisely blocking signaling pathways abnormally activated during OA progression. For example, excessive Hh pathway activation exacerbates cartilage degradation, and the κ-opioid receptor (KOR) agonist JT09 effectively inhibits Hh signaling and protects cartilage from degradation.^163^ Another key target is the ion channel Piezo1, a primary mechanosensor. Piezo1 activation directly induces chondrocyte apoptosis and ferroptosis. Therefore, blocking mechanically-triggered Ca^2+^ influx using the specific Piezo1 inhibitor Gsmtx4 effectively improves OA pathology.^136^ Studies on endogenous regulatory mechanisms reveal that G protein-coupled estrogen receptor (GPER) indirectly inhibits Piezo1 activation, providing important insights for developing new therapeutic targets.^164^

In preclinical OA and cartilage-engineering models, therapeutic modulation of mechanosensitive ion channels has generally followed two distinct strategies. Transient TRPV4 activation, particularly when combined with appropriate mechanical stimulation, has been explored to promote anabolic matrix synthesis and improve the integration of engineered cartilage constructs. In contrast, Piezo1 inhibition is intended to suppress overload-induced Ca^2+^ influx and downstream apoptotic or stress-associated signaling in chondrocytes. These findings support a context-dependent therapeutic model in which TRPV4 activity may be beneficial under controlled physiological loading, whereas excessive Piezo1 activation represents a target for protection against injurious mechanical stress.^165^^,^^166^

#### 4.2.2 Multi-target regulation: application of conventional drugs and natural products

In addition to targeting single pathways, many drugs with broad pharmacological activity show great potential. Some commonly used clinical drugs demonstrate new value in OA treatment. For example, the antidiabetic drug metformin significantly reduces matrix degradation and inflammatory markers in obese OA patients.^167^ Active components from natural products and dietary supplements have garnered significant attention, such as α-bisabolol (BISA). It simultaneously inhibits multiple inflammatory pathways including NF-κB, p38, and JNK, while undenatured type II collagen (UC-II) improves motor function and reduces systemic inflammation in patients.^168^^,^^169^ Meanwhile, traditional Chinese medicine provides a rich resource for OA treatment. Compound formulations such as Soufeng Sanjie Fang and Gu Bi Zhi Tong Fang demonstrate therapeutic potential in regulating immunity and metabolism through synergistic multi-component, multi-target effects.^170^, ^171^, ^172^

Unlike pharmacological interventions that primarily target intracellular signaling, biomaterials can modulate the extracellular mechanical and biochemical microenvironment simultaneously. Their therapeutic effects therefore depend not only on the active agents they carry, but also on material properties such as stiffness, viscoelasticity, stress relaxation, permeability, friction, degradation behavior, and responsiveness to joint stimuli. These properties determine the mechanical state and pathological exposure of chondrocytes and may consequently influence mechanosensitive signaling and downstream tissue responses.

### 4.3 Immunomodulatory biomaterials: mechanical regulation of the OA immune microenvironment

Macrophages are highly responsive to the physical and biomechanical properties of their local microenvironment. In OA, changes in cartilage and synovial tissue mechanics, including matrix softening during early degradation and fibrotic stiffening at later stages, may alter macrophage polarization and inflammatory activity.^173^ Biomaterials can therefore be designed not only to replace damaged cartilage matrix but also to actively regulate immune responses through stiffness, stress relaxation, pore architecture, surface topography, and degradation behavior. Piezo1 is upregulated in OA synovial macrophages and may promote mechanically induced M1 polarization, whereas Piezo1 knockdown attenuates OA progression.^174^ In addition, chondrocytes cultured on matrices with physiological stiffness produce extracellular vesicles that reduce cartilage inflammation and favor M1-to-M2 macrophage polarization; vesicles generated under pathological stiffness conditions exhibit more pro-inflammatory effects.^175^ Material architecture and degradation may further influence this response: porous and elastic structures can regulate macrophage behavior through cytoskeletal mechanotransduction, while rapid stress relaxation and degradable matrix components may facilitate a transition from early debris-clearing inflammation toward a reparative macrophage phenotype.^176^, ^177^, ^178^, ^179^

Beyond these intrinsic mechanical cues, biomaterials can provide localized and sustained delivery of immunomodulatory agents. Hydrogel- or particle-assisted delivery of mechanically conditioned exosomes may enhance their intra-articular retention and regulate chondrocyte-macrophage communication.^180^ Similarly, controlled release of Wnt inhibitors, antioxidant small molecules, nanozymes, or immunoregulatory ions may suppress ROS accumulation, inflammasome-associated activation, and M1 polarization while supporting matrix preservation.^181^^,^^182^ However, these delivery-based systems should be distinguished from materials that directly modulate the mechanical immune niche: their principal effect is biochemical regulation of inflammation or oxidative stress, although preservation of matrix integrity may secondarily improve the local mechanical environment.

### 4.4 Integrated biomaterial platforms for OA management

Hydrogels and nanomaterials have been widely investigated for OA treatment because their composition, mechanical properties, surface characteristics, degradation behavior, and drug-release profiles can be regulated to meet different therapeutic requirements. Hydrogels provide three-dimensional, water-rich polymer networks suitable for lubrication, structural support, and local delivery, whereas nanomaterials offer adjustable size, morphology, surface chemistry, and functional composition. These features also enable their integration into composite platforms that combine mechanical reinforcement, lubrication, controlled release, and biochemical regulation.

According to their principal therapeutic functions, these platforms can be grouped into materials that directly modify the mechanical and tribological environment and those that primarily provide drug delivery or pathological microenvironment regulation. The former are designed to provide mechanical support, ECM-mimetic viscoelasticity, stress relaxation, or reduced friction. The latter mainly improve local therapeutic exposure, scavenge ROS, modulate inflammation, or support tissue repair. Multifunctional systems may combine these functions within a single formulation. Representative hydrogel and nanomaterial applications are illustrated in Fig. 3, Fig. 4, respectively (see Fig. 5).

**Fig. 3:**
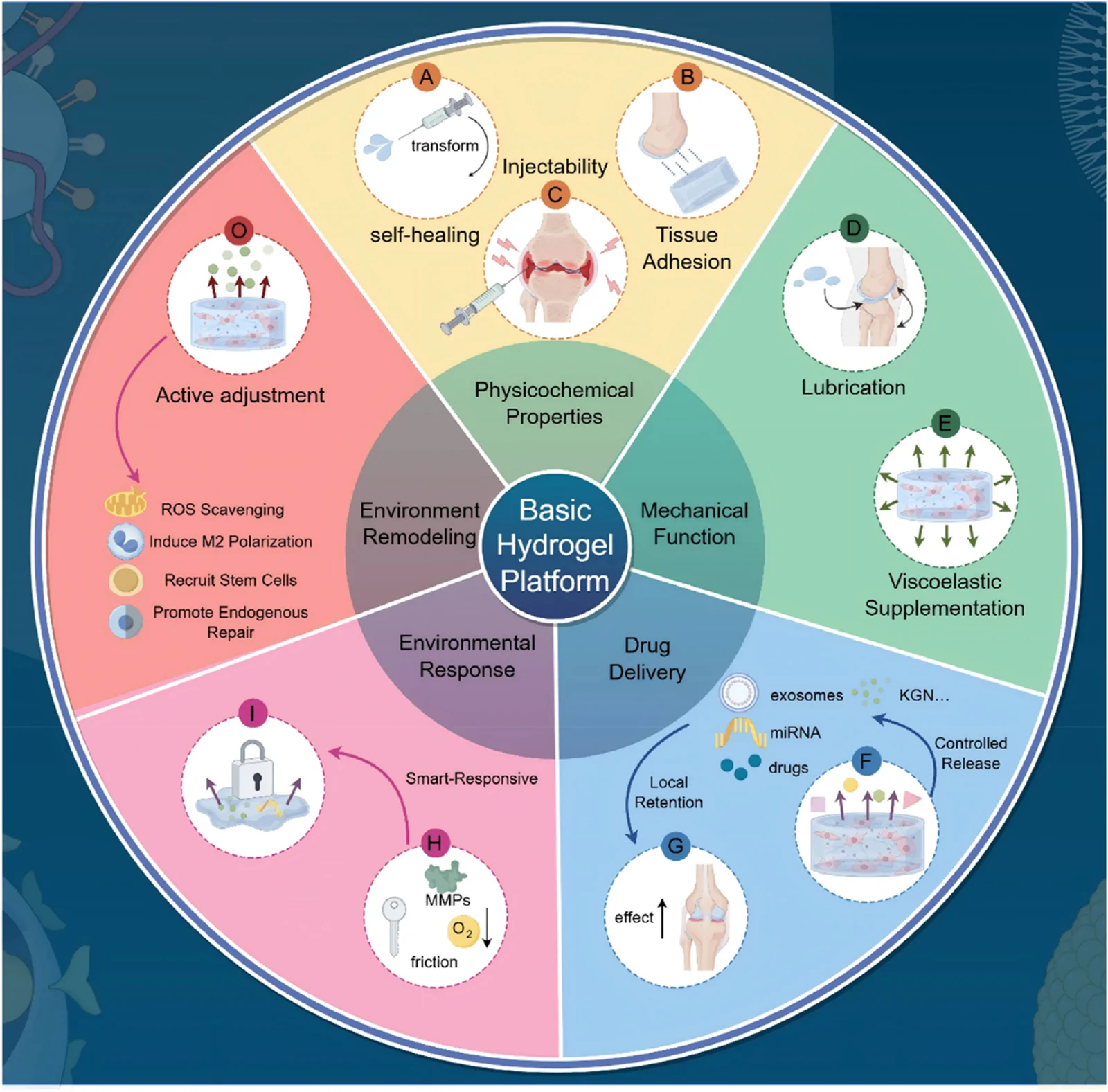
Application of multifunctional hydrogel technology in OA treatment.

**Fig. 4:**
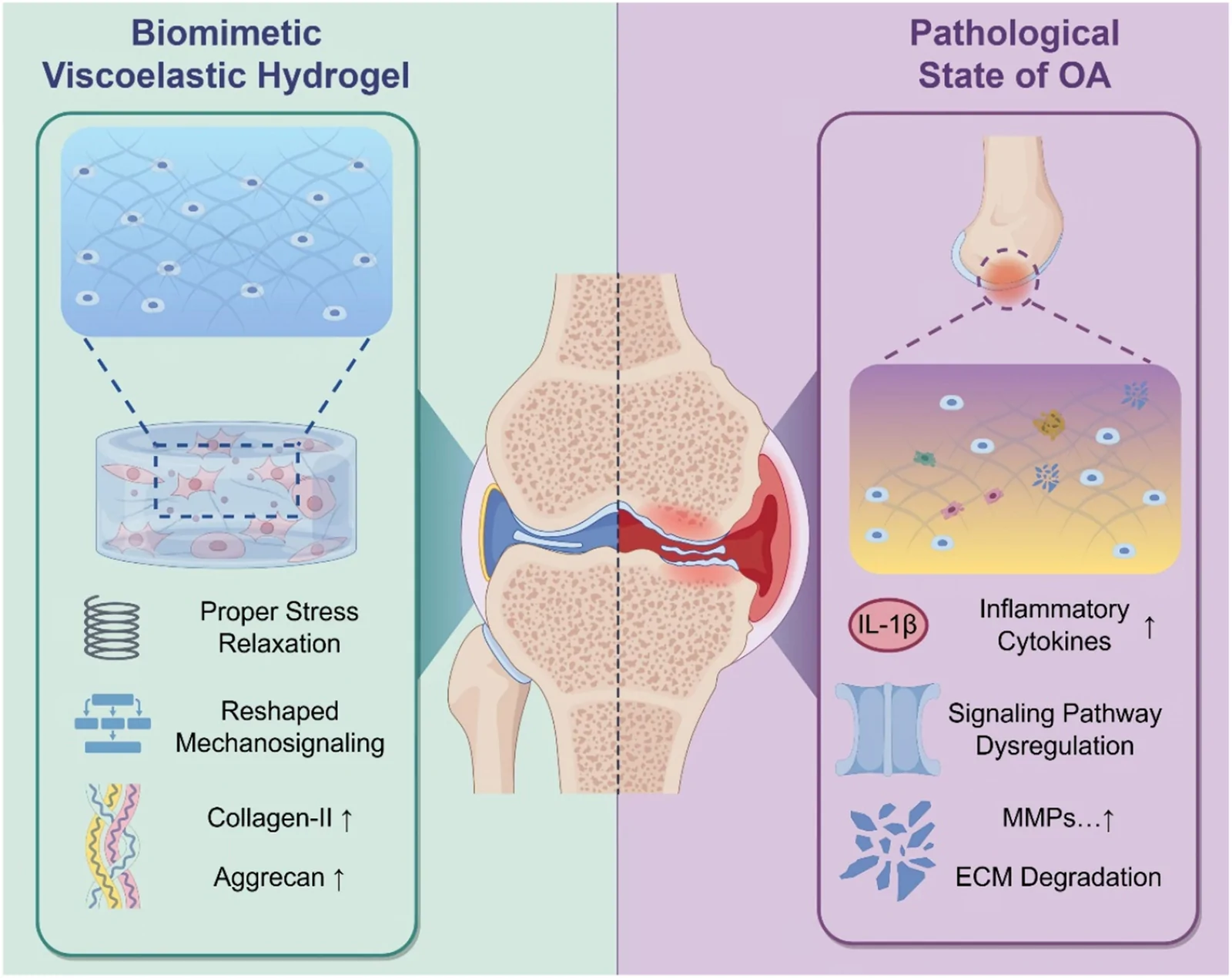
Biomimetic hydrogel therapeutic strategies based on matrix viscoelasticity regulation.

**Fig. 5:**
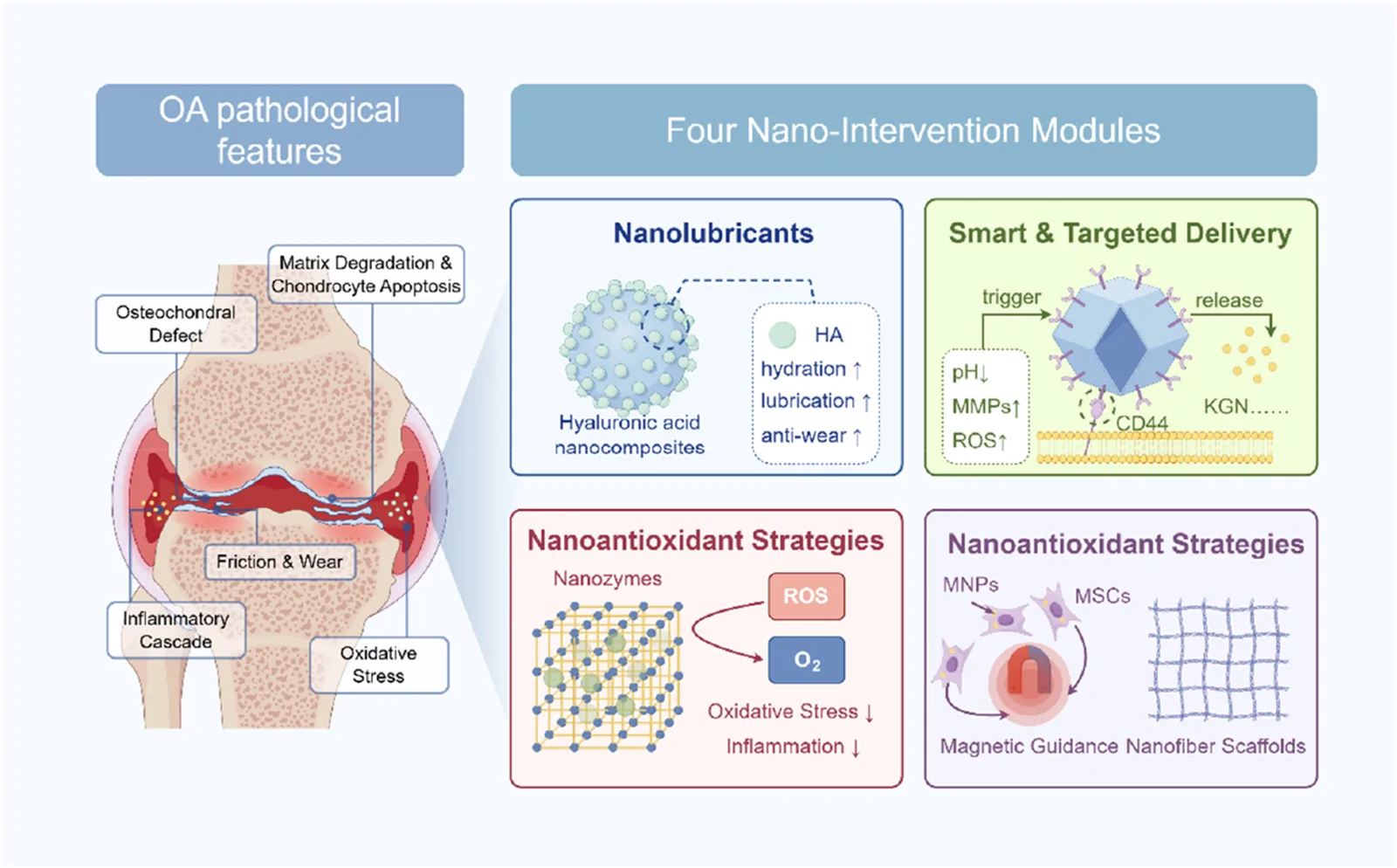
Nanotechnology application strategies in mechanical intervention for OA.

#### 4.4.1 Biomaterial platforms for direct mechanical and tribological modulation

##### 4.4.1.1 Mechanically reinforced and tissue-adhesive platforms

Biomaterials intended to provide local mechanical support must possess sufficient structural integrity and tissue retention under repeated joint loading. Injectable hydrogels with shear-thinning and self-healing properties can flow through a needle and recover their gel state after injection, allowing them to fill irregular cartilage defects.^183^, ^184^, ^185^ Dynamic covalent bonds, such as Schiff-base linkages, and supramolecular host–guest interactions are commonly used to achieve these properties.^183^^,^^186^

Mechanical reinforcement can be introduced through the incorporation of montmorillonite, graphene oxide, or other nanoscale components, as well as through interpenetrating polymer networks. These approaches improve network strength and resistance to deformation.^186^, ^187^, ^188^, ^189^ In addition, catechol-containing groups derived from dopamine enhance adhesion to wet cartilage surfaces and may reduce material loss during joint movement.^186^, ^187^, ^188^, ^189^ The combination of an injectable network, reinforcing components, and adhesive groups therefore provides a practical basis for defect filling and temporary tissue support.

The performance of these platforms depends on the balance among stiffness, permeability, and degradation. Increasing crosslinking density can improve mechanical stability but may restrict transport and tissue remodeling, whereas rapid degradation can shorten the duration of support. For repair-oriented scaffolds, degradation should also accommodate new matrix deposition while maintaining sufficient structural integrity. Lignin-based nanofibers and related composite materials offer adjustable mechanical, antioxidant, and structural properties for cartilage tissue engineering.^190^ However, their capacity to support stable, load-bearing tissue that integrates with host cartilage remains to be established.

##### 4.4.1.2 Viscoelastic and stress-relaxing platforms

Hydrogels and nanocomposite networks can be designed to reproduce selected time-dependent mechanical properties of cartilage (Fig. 4). Their viscoelasticity can be regulated by adjusting polymer concentration, crosslinking density, network architecture, and reinforcing components. Semi-interpenetrating and fully interpenetrating networks, together with lignin- or nanosheet-containing composites, have been used to modify stiffness, toughness, energy dissipation, and stress relaxation.^188^^,^^190^, ^191^, ^192^

Storage modulus, loss modulus, and stress-relaxation rate have been reported to influence chondrocyte morphology, proliferation, and ECM deposition.^188^^,^^191^^,^^193^ In particular, cellulose nanocrystal–collagen composite hydrogels with enhanced viscoelastic properties promoted chondrocyte proliferation and ECM deposition and were associated with FAK–YAP activation and reduced IL-1β expression.^191^ These findings illustrate how nanoscale reinforcement and hydrogel network design can be combined to provide a matrix environment that supports chondrocyte function.

Stress relaxation is a particularly relevant parameter for these platforms. Healthy chondrocytes exhibit enhanced functional responses in rapidly relaxing matrices, whereas OA chondrocytes show reduced sensitivity to this property.^194^ This difference may involve altered TRPV4–GSK3β signaling,^47^^,^^194^ suggesting that material performance may depend on the condition of the recipient cells. Related viscoelastic networks have also been used to promote chondrogenic differentiation in stem-cell-based systems.^188^^,^^190^^,^^195^

The design of these materials should balance cellular accommodation with durability under repeated loading. A high storage modulus may improve structural support without reproducing the relaxation behavior of native cartilage, while a rapidly relaxing network may accommodate remodeling but provide insufficient long-term support. In addition, changes in viscoelasticity frequently accompany changes in biochemical composition, ligand presentation, and adhesion. Their individual contributions to cellular outcomes therefore remain difficult to separate in some formulations.

##### 4.4.1.3 Lubricating hydrogels and nanolubricants

Lubricating platforms aim to reduce friction and wear at the cartilage surface through hydrated polymer networks or surface-associated nanoscale structures. Natural polymers, including hyaluronic acid and carrageenan, can be used to construct hydrogels with lubrication and viscoelastic properties^196^, ^197^, ^198^. More sophisticated systems reproduce selected self-lubricating features of cartilage or the meniscus by retaining water and releasing lubricating molecules during joint movement.^196^^,^^197^

Nanomaterial incorporation provides additional approaches for improving lubricant retention and surface coverage. HA-based nanocomposite systems have been developed using silica, gold, titanium dioxide, or calcium carbonate nanoparticles. Chemical modification, layered assembly, and physical adsorption can anchor HA or other hydrophilic polymers to nanoparticle surfaces, enhancing hydration and lubrication performance.^199^^,^^200^ These systems combine the lubricating properties of hydrated polymers with the adjustable surface characteristics of nanoparticles.

The effectiveness of lubricating platforms depends on surface hydration, coefficient of friction, permeability, and stability during cyclic loading. Friction-responsive formulations can further couple joint movement to lubricant release or hydration-layer recovery.^196^^,^^197^ By maintaining a hydrated interface and limiting cartilage surface damage, these materials may reduce shear-related loading and secondary tissue injury. However, improvements in friction and wear should be evaluated separately from direct changes in intracellular mechanotransduction.

##### 4.4.1.4 Multifunctional platforms combining lubrication and biochemical protection

Multifunctional platforms combine direct mechanical protection with regulation of oxidative stress or inflammation. Integrating these functions within a single hydrogel, nanoparticle, or nanocomposite formulation may help maintain lubrication while limiting the biochemical damage associated with cartilage wear.

Iron-doped ZIF-8 nanozyme hydrogels have been reported to scavenge ROS and provide oxygen during catalytic reactions. Their lubricating properties may additionally reduce frictional injury, producing combined effects on mechanical wear, oxidative stress, and tissue protection.^201^ Similarly, pyrogallic acid–manganese nanoparticle-containing systems have been associated with reduced oxidative damage, cartilage wear, and osteophyte formation in preclinical OA models.^202^

Surface-engineered nanoparticles provide another approach. Core–brush nanoparticles containing a polydopamine core offer ROS-scavenging activity, while their outer polymer brushes generate a hydrated lubricating layer.^203^ Hydrogel platforms carrying platelet-derived components can likewise combine lubrication with ROS reduction and modulation of macrophage inflammatory responses.^197^^,^^204^ These examples demonstrate that mechanical and biochemical functions can be incorporated through different structural components of the same material.

Combining several functions may also alter stability, degradation, drug release, and manufacturing reproducibility. The contribution of each component should therefore be distinguished when evaluating therapeutic outcomes, particularly where reduced friction, antioxidant activity, and delivered cargos may independently contribute to cartilage protection.

#### 4.4.2 Biomaterial platforms for drug delivery and pathological microenvironment regulation

##### 4.4.2.1 Injectable depots and sustained-release carriers

Conventional intra-articular injections often have limited therapeutic duration because drugs are rapidly diluted or cleared from the joint cavity.^205^ Injectable hydrogels address this limitation by forming a three-dimensional depot that increases local retention and enables sustained or controlled release. Shear-thinning, self-healing, and adhesive formulations facilitate administration and help maintain the depot at the intended site.^183^, ^184^, ^185^, ^186^, ^187^, ^188^, ^189^

Hydrogels have been used to prolong the intra-articular retention of corticosteroids, nonsteroidal anti-inflammatory drugs, and other therapeutic molecules.^187^^,^^196^^,^^205^ They can also carry simvastatin, kartogenin, miRNAs, exosomes, and platelet lysates to regulate inflammation, matrix metabolism, or tissue repair.^189^^,^^196^^,^^197^^,^^206^ Nanovesicles and other nanoscale carriers provide additional control over cargo encapsulation and local delivery. Representative cargos include KAFAK peptides, kartogenin, and other anti-inflammatory or cartilage-protective agents.^1^^,^^207^, ^208^, ^209^

The main design parameters for these platforms are loading capacity, release kinetics, permeability, degradation rate, and tissue retention. Hydrogel networks and nanoscale carriers can also be combined to provide complementary control over depot persistence and cargo delivery. Their therapeutic effects primarily depend on the activity and local availability of the delivered agents, while preservation of matrix integrity may secondarily improve the mechanical environment.

##### 4.4.2.2 Stimuli-responsive and targeted delivery platforms

Stimuli-responsive hydrogels and nanomaterials are designed to change their structure or release behavior in response to features of the OA joint. MMP-sensitive hydrogels can undergo enzymatic degradation in protease-rich environments, enabling local release of therapeutic agents.^196^^,^^204^^,^^206^^,^^210^ Other hydrogel systems respond to ROS, pH, hypoxia, or friction and may provide temporally regulated release.^196^^,^^204^^,^^206^^,^^210^ Similarly, ROS-responsive and pH-responsive nanocarriers use pathological biochemical conditions as endogenous release triggers.^211^^,^^212^

These approaches can be integrated with surface targeting to improve local therapeutic exposure. HA-containing systems may exploit interactions between HA and CD44 on chondrocytes or other joint-associated cells. KZIF@HA nanogels have been reported to support targeted delivery and cartilage regeneration in preclinical models.^213^ However, CD44 is expressed by several joint cell populations, and localization may vary with disease stage and tissue condition.

The performance of responsive and targeted platforms depends on stimulus availability, diffusion, clearance, and material degradation. Responsiveness demonstrated in vitro therefore requires confirmation under intra-articular conditions. For mechanically responsive formulations, the release profile should also remain reproducible across relevant loading magnitudes and frequencies. Where a platform combines responsive delivery with lubrication, these functions should be characterized independently.

##### 4.4.2.3 Antioxidant and immunomodulatory platforms

Hydrogels and nanomaterials can regulate the pathological microenvironment through intrinsic bioactivity or incorporation of functional components. ROS-scavenging hydrogels containing cerium oxide or other functionalized nanoparticles have been investigated to reduce oxidative stress and associated inflammatory and catabolic responses.^197^^,^^204^ Nanozymes and catalytic nanoparticles offer a related approach by reproducing or enhancing endogenous antioxidant activity. As illustrated by the multifunctional systems described above, these components can also be incorporated into lubricating networks.^201^^,^^202^

Drug loading and surface modification provide further opportunities for regulating macrophage inflammatory responses. Hydrogel formulations carrying bioactive components have been used to promote a less inflammatory local environment.^197^^,^^204^ These delivery-based applications complement the mechanically regulated immune responses discussed in Section 3.3, with material design focused on local retention, sustained activity, and appropriate exposure to immunomodulatory agents.

For antioxidant platforms, catalytic activity, tissue distribution, degradation products, and persistence are central considerations. For immunomodulatory delivery systems, cargo stability and release kinetics are additionally important. Their primary therapeutic contributions are reduced oxidative stress and inflammatory activity; restoration of cartilage mechanical properties or mechanical sensing requires separate evidence.

##### 4.4.2.4 Repair-oriented and externally controlled delivery platforms

Biomaterial platforms can support tissue repair by delivering regenerative factors, retaining therapeutic cells, or providing a three-dimensional niche for endogenous cell recruitment. Hydrogels have been investigated for delivering kartogenin, miRNAs, exosomes, and platelet-derived components, as well as for supporting cell proliferation and chondrogenic differentiation.^184^^,^^189^^,^^196^^,^^197^^,^^206^ Their hydrated networks provide space for cell incorporation, while adjustable adhesion and degradation help regulate retention and matrix formation.

Nanomaterials can introduce additional control into cell-based approaches. Magnetically responsive nanoparticles incorporated into mesenchymal stem cell systems allow external magnetic fields to influence cell aggregation, localization, or differentiation.^214^ This provides a potential means of coordinating cell delivery with external physical stimulation. The resulting performance depends on particle–cell interactions, applied-field conditions, and the compatibility of the delivery platform with the surrounding tissue.

These repair-oriented strategies are supported mainly by in vitro and animal studies. Increased cell recruitment, chondrogenic marker expression, or matrix deposition does not necessarily establish the formation of mechanically functional cartilage. Further evaluation should determine whether the newly formed tissue integrates with host cartilage and maintains its properties under sustained joint loading.

The translation of integrated biomaterial platforms depends on matching material performance to the intended therapeutic function. Platforms for mechanical protection must retain their structural and lubricating properties under repetitive loading, whereas delivery platforms require sufficient retention and predictable therapeutic exposure. These requirements may conflict: increased crosslinking can improve mechanical stability while reducing permeability and slowing release, whereas rapid degradation may facilitate delivery but shorten the duration of support.

Joint motion, synovial clearance, protein adsorption, enzymatic activity, and inflammation can alter material persistence and performance. Additional considerations include the safety of nanoparticles and degradation products, potential synovial reactions, systemic distribution, and long-term accumulation. Composition, fabrication, sterilization, storage, and injection conditions also require standardization to ensure reproducibility.

Most advanced hydrogel, nanomaterial, and composite platforms remain at the preclinical stage. Tribological studies establish friction and wear performance, cell studies assess biological responses, and ex vivo or animal models provide evidence within more complex mechanical and tissue environments. Clinical translation will require these findings to be connected with durable structural and functional outcomes. Representative platforms, material design parameters, mechanobiological targets, experimental models, and evidence levels are summarized in Table 3.

**Table 3:** Representative hydrogel- and nanomaterial-based strategies for modulating mechanical and pathological microenvironments in osteoarthritis.

Strategy	Representative material	Key design parameters	Primary function	Mechanobiological target/evidence	Model and evidence level
Injectable drug-depot hydrogel	Corticosteroid-loaded gellan sheared hydrogel	Shear-thinning viscoelastic network; self-restructuring; sustained release (17-18 d)	Local anti-inflammatory drug delivery	Synovial inflammatory microenvironment; direct mechanotransduction not assessed	Primary human joint-associated cells; TNFtg mice; in vitro + small-animal preclinical
Adhesive or cell-delivery hydrogel	RGD-modified OHA/ADH-HAMA hydrogel carrying ASA and BMSCs	Dynamic hydrazone/photocrosslinking; self-healing; G′ approximately 1 kPa	Defect filling, cell delivery, osteo-/chondrogenic support, and immunomodulation	Cell-ECM adhesion, BMSC spreading, and macrophage polarization; direct mechanotransduction pathway evidence not assessed	BMSC/macrophage culture; rat defect model; in vitro + small-animal preclinical
ECM-mimetic cartilage scaffold	dECM-SELP cartilage patch	Decellularized cartilage architecture; porous viscoelastic adhesive network	Structural support and cartilage regeneration	ECM-like load-bearing niche; chondrocyte adhesion, phenotype maintenance, and matrix synthesis; direct pathway evidence not assessed	Equine chondrocyte culture; in vitro preclinical
Viscoelastic stress-relaxing hydrogel	CNC/collagen microporous hydrogel	G’ 10-30 kPa; stress relaxation 29-261 s; micropores 195-222 um	Matrix viscoelasticity-based regulation of chondrocyte behavior	Cell spreading, ECM deposition, and inflammatory response; FAK/YAP localization associated with stress relaxation	Primary rat chondrocytes; in vitro preclinical
Lubricating/viscosupplement hydrogel	Gellan gum-HA hydrogel	Shear-thinning; viscoelastic; hyaluronidase resistance; injectable	Restoration of synovial viscoelasticity and lubrication	Friction and shear reduction; direct cellular mechanotransduction not assessed	Tribological testing; chondrocyte culture; in vitro/ex vivo evidence
Friction-responsive regenerative hydrogel	DS/KGN nanoliposome-gelatin hydrogel	Injectable; friction-responsive release; hydration-layer reassembly; fatigue resistance	Self-lubrication, controlled release, and cartilage repair	Meniscal friction and cartilage shear protection; friction-triggered release directly assessed, intracellular mechanotransduction not assessed	Rat meniscal injury model; small-animal preclinical
Immunomodulatory self-lubricating hydrogel	Platelet lysate-loaded PNAAA hydrogel	Shear-thinning/self-healing; hydration lubrication; sustained protein release	Lubrication, ROS reduction, and immunomodulation	Frictional shear and inflammatory niche; M1-to-M2 polarization and noncanonical NF-kB inhibition; force-sensing channels not assessed	Chondrocyte/macrophage culture; ACLT rat OA; in vitro + small-animal preclinical
Stimuli-responsive therapeutic hydrogel	TGF-β/KGN-loaded GelMA/F127DA hydrogel	MMP-responsive degradation; compression-triggered release; photocrosslinking	Sequential recruitment, chondrogenesis, and anti-inflammatory delivery	OA protease-rich niche and joint compression; direct intracellular mechanotransduction not assessed	BMSC culture; rat osteochondral-defect OA; in vitro + small-animal preclinical
Antioxidant/lubricating nanocomposite hydrogel	PGA-Mn-loaded oxidized alginate-gelatin hydrogel or Fe/ZIF-8/Gel hydrogel	Nanoscale antioxidant component; compressive modulus; low friction or catalytic ROS-scavenging capacity; sustained release	Combined lubrication and oxidative-stress regulation	Friction-wear-ROS-inflammatory feedback; direct mechanotransduction not assessed	Chondrocyte culture; ACLT or ACLT + MMx rat OA; in vitro + small-animal preclinical
Multifunctional lubricating nanoparticle	Curcumin-conjugated SiO2@PDA@PMPC-PBA core-brush nanoparticle	Approximately 140 nm; hydration brush; ROS-cleavable linkage; friction coefficient 0.15	Hydration lubrication, ROS-responsive drug delivery, and immunomodulation	Frictional shear and macrophage inflammatory niche; M1-to-M2 polarization via Nrf2/HO-1/NQO-1; direct force-sensing pathways not assessed	Macrophage culture; MIA rat OA; in vitro + small-animal preclinical
Clinical viscosupplementation	Cross-linked high-molecular-weight HA and sodium hyaluronate formulations	Molecular weight/crosslinking-dependent elastoviscosity; single or repeated injection	Symptomatic lubrication and load buffering	Joint friction and nociceptive mechanical input; direct mechanotransduction not assessed	Human RCTs, cohorts, and meta-analyses; clinical evidence

## 5 Emerging research perspectives

The onset and development of OA involve not only cartilage degeneration but also interactions between multiple tissues at mechanical and biological levels. To this end, researchers have embarked on studies from multiple emerging perspectives to gain comprehensive understanding of OA pathological mechanisms and develop new treatment strategies.

### 5.1 Multi-tissue interaction model research

OA occurrence and development involve not only cartilage degeneration but also interactions between bone, cartilage, synovium, and other tissues at mechanical and biological levels. To this end, researchers have developed numerous in vitro research models.^215^, ^216^, ^217^ For example, cartilage-bone-synovium co-culture models are used to investigate post-traumatic OA (PTOA) mechanisms. This model simulates early PTOA pathological processes and reveals that mechanical injury and inflammation play key roles in early PTOA progression.^215^ Additionally, models simulating the interface between articular cartilage and subchondral bone have successfully reproduced OA progression under both healthy and pathological conditions, providing tools for studying OA development and mechanical and biological interactions between multiple tissues.^216^

Furthermore, computer-based simulation models target macroscopic stress. Some studies have utilized convolutional neural networks (CNNs) to provide automated cartilage segmentation methods.^218^ Others have employed multi-scale computer modeling methods to assess cartilage mechanical stress differences during walking between healthy individuals and early KOA patients.^219^ For micro-level regulatory networks, a study constructed a chondrocyte mechanical sensitivity network model comprising 118 mechanosensitive nodes and 358 directed edges to simulate mechanical stress and inflammation effects on chondrocyte gene expression.^108^ These approaches provide methods for understanding OA underlying mechanisms and developing novel therapies.

### 5.2 The role of vascular and neural invasion

Normal articular cartilage does not contain blood vessels, nerves, or lymphatics, which facilitates its role in stress buffering.^220^ However, the abnormal stress environment in TMJOA leads to microenvironment formation that induces vascular and neural invasion in cartilage.^221^ Specifically, pro-angiogenic factors secreted by macrophages and chondrocytes, along with hypoxia-induced HIF-1α, promote angiogenesis. Molecules involved in angiogenesis, such as semaphorins, neuropilins, and plexins, regulate neural invasion, leading to persistent burning pain.^222^^,^^223^ Neurovascular invasion is closely associated with cartilage inflammation: angiogenesis promotes inflammation during endochondral ossification, leading to structural lesions. Vascular-induced neural growth exacerbates inflammation, disrupts the osteochondral junction, and results in inappropriate neural innervation of joint structures, thereby worsening pain.^223^ These phenomena suggest that neurovascular invasion may be a key pathological process in abnormal stress-induced cartilage damage.

### 5.3 Effects on the immune system

In the immune system, T cells play a key role in mechanical stress-induced joint damage and OA progression. In mechanical stress models, T cell numbers in local lymph nodes significantly increase with prolonged loading time. In mice with T cell receptor alpha deficiency (*TCRα*^−/−^), mechanical stress-induced cartilage degeneration and osteophyte formation are reduced. Inhibiting T cell migration from lymph nodes alleviates cartilage degradation.^224^ Regulating T cell migration may emerge as a therapeutic strategy for OA. Reducing mechanical stress alleviates cartilage damage, subchondral bone changes, and secondary inflammation in OA joints, providing a theoretical basis for modulating the mechanical environment to influence immune responses.^225^

Additionally, interactions between immune cells and inflammatory factor regulation significantly impact OA progression.^226^ The polarization state of synovial macrophages (M1/M2) is closely associated with OA inflammation severity. Mechanical stress can modulate the PI3K/AKT/NF-κB signaling pathway, altering the M1/M2 ratio of synovial macrophages, reducing M1 (pro-inflammatory) macrophages, and increasing M2 (anti-inflammatory) macrophages, thereby alleviating inflammatory responses and protecting cartilage.^22^

## 6 Conclusions

The progression of osteoarthritis (OA) is primarily driven by mechanical imbalance. Chondrocytes act as key mechanosensors, converting abnormal mechanical signals into pathological responses. Excessive mechanical stress activates mechanosensitive ion channels (e.g., Piezo1, TRPV4) and initiates key signaling pathways (e.g., NF-κB, Wnt, YAP). This activation promotes inflammation, cell death, senescence, and extracellular matrix degradation. These events establish a vicious cycle that accelerates OA progression.In contrast, moderate mechanical stimuli activate protective pathways (e.g., TGF-β/Smad2/3, SIRT, PI3K/Akt). These pathways enhance matrix synthesis, suppress inflammation, and inhibit apoptosis, thereby maintaining cartilage homeostasis and delaying degeneration. This dual response highlights the central role of mechanical signaling in OA pathophysiology and therapeutic strategies.

Therefore, future OA therapies should target not only symptom management but also the modulation of chondrocyte mechanobiological responses. At the macro level, interventions such as exercise therapy and surgical procedures can restore overall mechanical balance. At the micro level, approaches including targeted signaling modulation and biomimetic materials (e.g., hydrogels, nanomaterials) optimize the cellular mechanical environment. These evolving strategies represent a transition in OA treatment from “mechanobiology” to “mechanomedicine.” Ultimately, an integrated strategy that addresses both macro- and micro-mechanical environments may support disease modification and joint preservation. However, whether these approaches can produce durable structural repair or reverse OA progression in humans remains to be established in rigorously designed clinical studies.

## CRediT authorship contribution statement

**Ziqi Gao:** Writing – original draft, Visualization, Project administration, Data curation. **Yule Zheng:** Writing – original draft. **Hongxiang Mei:** Writing – review & editing, Validation, Supervision. **Juan Li:** Writing – review & editing, Supervision, Project administration, Funding acquisition.

## Ethical approval

This manuscript is a review article. It does not contain any original research involving human participants, human specimens, human data, or animals conducted by any of the authors. Accordingly, ethical approval was not required for this study, and informed consent was not applicable.

## Declaration of competing interest

The authors declare that they have no known competing financial interests or personal relationships that could have appeared to influence the work reported in this paper.
